# Desorption Electrospray Ionization–Mass Spectrometry Imaging Provides Spatiochemical Information on Potential Biocontrol Agents against *Phytophthora capsici* Infection in Tomato Plants

**DOI:** 10.1021/jasms.6c00067

**Published:** 2026-05-07

**Authors:** Jamille Y. Robinson, Hawkins S. Shepard, Daniel Ambachew, Peter J. Eyegheleme, Jody C. May, Margaret T. Mmbaga, John A. McLean

**Affiliations:** † Department of Agricultural & Environmental Sciences, 5717 Tennessee State University, Nashville, Tennessee 37209, United States; ‡ Department of Chemistry, Center for Innovative Technology, 5718 Vanderbilt University, Nashville, Tennessee 37235, United States

**Keywords:** DESI-MS, mass spectrometry imaging, oomycete, molecular engineering, spatial lipidomics, biocontrol

## Abstract

Biological control agents can offer an eco-friendly and more sustainable alternative to conventional chemical pesticides, providing protection against destructive pathogens, such as *Phytophthora capsici*, while reducing potential environmental harm associated with synthetic pesticide use in agricultural systems. This work evaluates the biocontrol effectiveness of *Bacillus vallismortis*, *Bacillus amyloliquefaciens*, *Bacillus thuringiensis*, and *Bacillus subtilis*, against the widespread plant pathogen *Phytophthora capsici*. Our studies showed that *Bacillus thuringiensis* and *Bacillus subtilis* promote plant growth and provide protection against *Phytophthora capsici* in both in vitro and in vivo greenhouse studies, while *Bacillus vallismortis* and *Bacillus amyloliquefaciens* were effective in vitro but not in vivo. Specifically, *Bacillus thuringiensis* was observed to both hinder the growth of *Phytophthora capsici* and enhance plant resilience to this pathogen, with *B. thuringiensis* *-*treated, pathogen-exposed plants displaying a 94.4% increase in root length and a 74.0% increase in shoot height compared to plants with only oomycete exposure. To probe the molecular interactions between the biocontrol agent and pathogen, a dual culture of *Bacillus thuringiensis* and *Phytophthora capsici* was analyzed in situ using a desorption electrospray ionization–mass spectrometry imaging (DESI-MSI) workflow. This approach interrogated the spatially oriented biochemical interactions that may serve as the molecular foundation for the effectiveness of these biological control agents in crop protection, identifying seven unique phenotypic regions within the dual culture. Herein, we demonstrate the benefits of biological control agent application in tomato cultivation and showcase the strengths of desorption electrospray ionization–mass spectrometry imaging when applied to the spatially resolved molecular characterization of agriculturally relevant microorganisms.

## Graphical abstract

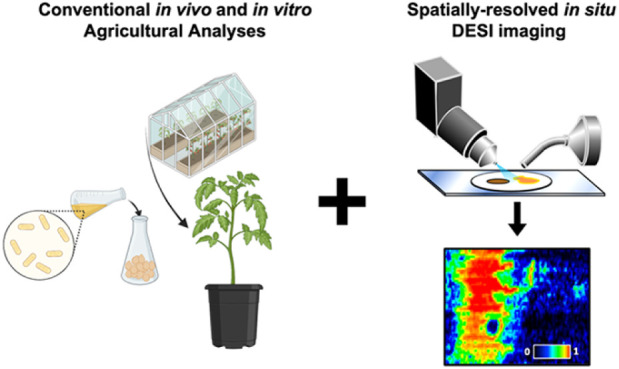

## Introduction

*Phytophthora capsici* is a notorious filamentous oomycete plant pathogen that causes significant harm in tomato production, primarily through root rot, fruit rot, and foliage blight. *Phytophthora spp*. primarily release effector proteins through specialized structures called haustoria that are used for infection establishment and absorbing nutrients from the host plant. The effector proteins hamper the plant’s ability to absorb water and nutrients, leading to stunted growth and vigor.^1,2^ Considering that *Phytophthora spp*. are the causative agents responsible for some of the most destructive plant diseases, their notoriety makes these organisms excellent models for studying the complex molecular interactions between plants and invading pathogens.

Historically, chemical pesticides have been widely used to control oomycete plant pathogens. However, synthetic pesticides have several shortcomings, such as soil contamination attributed to accumulating chemical residues. These chemicals disrupt the soil microbiome by reducing microbial diversity and altering community structures, impairing essential soil functions. Furthermore, the selective pressure from pesticides fosters the development of resistant pathogen strains, exacerbating pest control challenges and potentially increasing pathogen virulence.^3,4^ Together, these effects contribute to a cycle of declining soil health, increased dependency on chemical inputs, and escalating environmental and agricultural issues. One promising alternative is the use of beneficial bacteria acting as biocontrol agents (BCA), which offer several advantages over traditional chemical pesticides. Specifically, root-dwelling, plant growth-promoting bacteria (PGPB) protect plants from diseases through a combination of competitive exclusion, production of antimicrobial compounds, enhancement of plant immune responses, and beneficial modifications of the rhizosphere environment.^5,6^ These mechanisms not only help in controlling pathogenic microbes but also contribute to overall plant health and productivity.

Many PGPBs produce antimicrobial substances, such as antibiotics and lytic enzymes, that inhibit or kill pathogenic microbes.^7^ These compounds can directly suppress or kill pathogens, reducing their ability to infect the plant. PGPBs can also trigger a plant’s defense mechanisms through induced systemic resistance (ISR). When plants detect specific signals from PGPB, they activate a wide range of defense responses that enhance their ability to fend off pathogens. These responses include the production of defensive chemicals, strengthening cell walls, and activating stress-related proteins.^8^ Additionally, PGPB can prime the plant immune system, making it more responsive to pathogen attacks. This priming induces a heightened state of alertness that allows the plant to respond more quickly and robustly when pathogens attempt to invade. Another protection facet is the ability of PGPB to preemptively occupy and colonize plant root surfaces and internal tissues. This ability can prevent pathogenic microbes from establishing themselves by forming a physical barrier that reduces the chances of infection by pathogens. The PGPB can also alter the rhizosphere’s chemical and physical properties in ways unfavorable to pathogens by modifying pH levels, producing volatile organic compounds, or altering the availability of certain nutrients, making the environment less conducive to pathogen survival and growth.^8,9^

*Bacillus* species play a significant role in the biocontrol of plant pathogens through various mechanisms. These beneficial bacteria are known for producing a wide range of bioactive compounds and adapting to different environmental conditions.^10–12^ In this work, in vitro studies examined the biocontrol effectiveness of *Bacillus spp.* [*B. vallismortis* (Ps), *B. amyloliquefaciens* (Psl), and *B. thuringiensis* (IMC8)] against *P. capsici* in the management of tomato diseases. The results indicated a reduction in mycelial growth attributed to *B. amyloliquefaciens* and *B. vallismortis*, while in vivo investigations demonstrated a greater reduction in disease severity compared to the systemic fungicide metalaxyl.^13^ While some PGPB have demonstrated the potential to serve as biocontrol vectors, strategies that determine the ecological functions of bacterial secondary metabolites, the variables that regulate their production, and their physicochemical properties are all still in their infancy.^14^ Substituting synthetic fungicides with bacterial BCAs in plant pathogen management is a viable and auspicious strategy, owing to its ecological impacts, diminished residues on agricultural commodities and the environment, and adaptability across diverse agricultural systems.^15,16^ However, before these alternatives can be implemented, further investigation and analysis of the potential mechanism of action is warranted to determine more effective ways to elucidate the metabolome and underlying biochemical processes of BCA-pathogen interactions.

Mass spectrometry (MS) based workflows are frequently implemented in biochemical profiling due to the ability to leverage the high sensitivity, specificity, and speed of MS toward the study of small molecules via metabolomic and lipidomic analyses.^17^ Mass spectrometry imaging (MSI) approaches are able to garner spatiochemical information during location-resolved sampling, allowing for the creation of molecular maps that inventory the chemical signals detected within a sampling region. These techniques go beyond mere detection and provide important insight into the distribution of specific analytes found within the samples. MSI-based workflows are increasingly utilized in medical fields, imaging tissue sections to characterize disease states, identifying the localization of exogenous compounds, and even providing quantitative data for the concentrations of biomolecules.^18–21^ Among the different implementations of MSI, desorption electrospray ionization (DESI) shows particular promise as it combines the benefits of traditional spray ionization methods with the analysis of solid phase samples usually associated with desorption-based approaches.^22^ Importantly, DESI experiments can be conducted under ambient conditions, allowing for the direct examination of living biological systems while minimally affecting the analysis of the small molecules required for effective characterization.^23^

This study investigated the feasibility of using DESI to examine agricultural biological systems involving biological control of plant pathogens, such as *P. capsici* using bacterial endophytes including *B. thuringiensis*. A coculture of *B. thuringiensis* and *P. capsici* was evaluated using DESI-MSI for the spatial analysis of chemical signals present in the BCA-pathogen system. We compared the effect of specific BCAs on *P. capsici* in tomato plants and found that *B. thuringiensis* exhibited the greatest effects in reducing disease severity and improving plant growth, prompting further characterization of potential mechanisms of action by coculturing the two organisms. DESI MSI allowed us to directly examine the living coculture grown on a membrane scaffold in situ and provide spatially resolved chemical profiles relating to this interspecies interaction. This study combined traditional agricultural characterization methodologies with DESI-MSI, and findings reported herein focused on specific BCA interactions between *B. thuringiensis* and *P. capsici* thus providing a potential workflow that can be used for the molecular characterization of agricultural biological control systems.

## Methods

### BCA and Pathogen Culturing

All bacterial strains used in this study are listed in Supplementary Table 1. Single colonies of endophytic *Bacillus* isolates were routinely cultured in Luria–Bertani (LB) broth and incubated at 30 °C for 16–20 h with aeration at 200 rpm. Cultures were adjusted to an optical density at 600 nm 1.0, which corresponds to approximately 10^8^ CFU/mL. Cultures were subsequently streaked on LB agar plates and incubated at 30 °C for 16 h for short-term storage or used directly for seed treatment. *P. capsici* was grown from 5 mm plugs (stock stored in distilled sterile water at room temperature) on V8 juice agar (20% V8 juice, 0.2% CaCO_3_, 1.5% Agar) at room temperature in the dark for 5–7 days. Zoospore suspensions were created by saturating the plates with cold sterile distilled water and carefully extracting the mycelial surface. The concentration was adjusted to 10^4^ zoospores CFU/mL. Dual culture studies used a 5 mm V8 agar plug of actively growing *P. capsici* placed at the center half of a 70 mm Petri dish containing a 50/50 mix of LB/V8.

### Evaluation of Biocontrol Bacterial Isolate Antagonism of *Phytophthora capsici*

To test the effects of BCA on plant growth and protection against *P. capsici* pathogen, tomato seeds (*Solanum lycopersicum* L. “Rutgers”) were surface sterilized in 1% sodium hypochlorite for 10 min and rinsed three times for 60 s in double distilled water until the smell of bleach was no longer present. Seeds were blotted dry using sterilized paper towels and placed in 50 mL conical tubes containing 30 mL of selected BCA bacterial suspension of 10^8^ CFU/mL for 24 h at 4 °C. Control treatments were comprised of tomato seeds soaked in sterile LB broth with no bacteria. Seeds were air-dried in a sterile laminar hood for 2 h prior to being sown in heat-sterilized Miracle-Gro potting mix in 9 cm containers, with a replication of seven individual plants per treatment and arranged in a randomized design. Studies were carried out in both growth chambers set at 26 ± 3 °C using heat-sterilized soil and in greenhouse environment using nonsterile soil.

In order to produce pathogen-infested soil, plants were drenched at the soil line with a liquid suspension of *P. capsici* zoospores at a concentration of 10^4^ spores per mL, harvested from actively growing *P. capsici* mycelia. Plants were BCA treated as outlined, maintained in a growth chamber for 6 weeks, and then moved to the greenhouse environment, where they were maintained for an additional four weeks (10 weeks total), after which plant biomass was measured by root and shoot dry weights, as well as total dry weight. Plant growth-promoting activity and pathogen protection were evaluated on Murashige and Skoog agar (Murashige and Skoog basal media; Sigma-Aldrich, 0.03% phytagel, pH 5.7) culture plates (11 × 11 cm square). Two 5 mm plugs of actively growing *P. capsici* were placed equal distances apart approximately 35 mm from the bottom of the plate. Plant growth was measured by root and shoot height of 7 day and 14 day-old seedlings using ImageJ software which was used to convert pixels to millimeters in optical images.

### Dual Culture In Vitro Microscopic Analysis

A 5 mm agar plug containing actively growing *P. capsici* mycelium was positioned at the midpoint of a 50/50 V8/LB agar plate. The plug was given 48 h to grow before streaking 20 mL of 10^8^ CFU/mL bacterial media roughly 3 cm away from the edge of the mycelial plug. The plates were placed in a dark environment at ambient temperature and monitored for 72 h. A dissecting microscope (Olympus XZS-ILLD2) coupled with an HD 4K camera was used to measure and analyze the inhibition zone and assess antagonistic activity between the BCAs and *P. capsici*.

### DESI Sample Preparation and Acquisition Settings

To prepare cocultures for DESI-MSI analysis, a 5 mm plug of *P. capsici* was placed on top of a nylon membrane set atop a 50/50: LB:V8 agar base. Subsequently, the same membrane was streaked with *B. thuringiensis*, and the coculture was allowed to incubate at room temperature in low light conditions for 5 days. Three separate cocultures were prepared and subjected to replicate DESI-MSI analyses, resulting in three biological replicates each analyzed on different days. Nylon membranes used in this work had a porosity of 0.22 μm and were 47 mm in diameter (Sigma-Aldrich, Z290807). Prior to imaging, the membrane and coculture were removed from agar and cut to the desired size using Mayo scissors. Once cut, the membrane and bacteria were allowed to dry for 5 min and then adhered to a glass microscope slide via double-sided Scotch tape. The mounted membrane was then imaged directly.

Imaging experiments were carried out on a Synapt G2-S High Definition Mass Spectrometer (Waters Corporation) fitted with a prototype DESI-MSI probe.^24^ Optimized DESI source parameters were found to be a capillary voltage of −5 kV, 150 °C source temperature, 0.45 bar of N_2_ gas flow, and an ablation diameter of 0.7 mm. DESI geometry was set to an x, y, z positional orientation of −2, +2, +2.75, respectively, and a sprayer angle of 70°. The DESI ionization solvent consisted of 90/10 acetonitrile/water, 0.2 ng/μL leucine-enkephalin, and 0.1% ammonium hydroxide. Pixel dimensions of 100 μm × 200 μm were used with a raster rate of 150 μm/sec. The instrument was mass calibrated with sodium formate salt clusters to a 95% confidence band and root-mean-square (RMS) residual mass of <0.5 ppm. All acquisitions were carried out in the mass range of *m*/*z* 50–1200, with experiments being performed in negative ion polarity mode.

### Data Processing and Spatiochemical Analysis

All raw imaging files were processed using HDImaging software (Waters), with the 4000 most abundant features present within the MSI being investigated. Lock mass adjustments were performed using the leucine-enkephalin internal standard. All subsequent spatial analysis on processed files was performed in R Studio (RStudio Team 2024, v1.1.383; R version 3.3.3). Feature intensities were normalized to the total ion chromatogram and then normalized to the leucine-enkephalin internal standard to account for sprayer inconsistencies and variation in sample morphology. Features were peak picked using a 10 ppm window. Spatially normalized heat maps were produced by plotting the intensity associated with peak picked features for each pixel location. Prior to segmentation, the initial number of families (k) and the shrinkage parameter (s) were determined empirically for experiments.^25^ Unsupervised segmentation was achieved using spatial shrunken centroids (SSC) analysis via the Cardinal MSI package, allowing for the identification of distinct spatiochemical regions within the coculture.^26^ Tentative identifications for features were generated by searching measured *m*/*z* values against the Kyoto Encyclopedia of Genes and Genomes (KEGG), METLIN, LipidMaps, and ChemSpider. A threshold of 10 ppm mass accuracy and 80% isotopic similarity were used for all tentative identifications.

## Results and Discussion

### BCA-Treated Plants Exhibit Growth Promotion and Pathogen Protection

There is increasing interest in the suppression of *P. capsici* on tomatoes and other solanaceous species and cucurbits with rhizobacteria. Previous studies have shown that isolates of *B. amyloliquefaciens* significantly promoted the growth of seedlings and reduced pathogen load on roots, suggesting broad spectrum antifungal biocontrol traits.^27^ *B. thuringiensis* is commonly used as a biocontrol agent to combat insect pests. However, new studies have revealed its potential as a preventive bacterial biocontrol agent for controlling *P. capsici* infections in tomato plants based on its antimicrobial compound production, competition with the pathogen for nutrients and space in the rhizosphere, and induction of tomato systemic resistance.^27–29^

Our analyses identified two BCA isolates, *B. thuringiensis* (IMC8) and *B. subtilis* (Prt), as growth promoting strains for tomato seedlings 3 weeks after treatment (Figure 1B,C). The BCA-treated plants exhibited similar growth characteristics when exposed to *P. capsici* with biomasses similar to control plants not inoculated with *P. capsici* and heavier than *P. capsici*-treated tomatoes (Figure 1D). Non-BCA-treated plants exposed to *P. capsici* exhibited a considerable decrease in biomass marked by increased infection and only 38% of plants survived after 10 weeks. Meanwhile, BCA-treated plants exposed to *P. capsici* had a 75% survival rate for IMC8 and Prt (Figure 1E). Plants treated with *B. amyloliquefaciens* (Psl) and *B. vallismortis* (PS) in the presence of *P. capsici* had a 38% and 25% survival rate, respectively. This indicated that IMC8 and Prt BCAs provided superior plant protection against *P. capsici* compared to PS and Psl. PS-treated plants demonstrated an 88% survival rate. Notably, plants treated with IMC8, Prt, and Psl with no pathogen exposure displayed 100% survival after 10 weeks, suggesting that BCA treatment could be offering additional protection from other potential pathogens present in nonsterile soil used in the greenhouse, considering that only 50% of non-BCA-treated control plants remained viable after 10 weeks (Figure 1E). However, in vitro studies revealed that all BCA-treated seedlings exhibited pathogen protection and growth promotion (Figure 2). This observation suggests that IMC8 both promoted plant growth and aided plant protection against *P. capsici*, with the overall plant weight being significantly heavier than the control not treated with BCAs. *Bacillus* isolates Prt, IMC8, and Psl all have statistically significant (p-value <0.05, 95% confidence interval) growth-promoting effects on tomato shoots in vitro (Figure 2C) compared to control after 2 weeks, but at 10 weeks of greenhouse study in the presence of the pathogen, IMC8 exhibited the most biomass increase among BCA-treated tomato seedlings in comparison to the nontreated control (Figure 1D). This suggested that growth promotion and disease protection may depend on factors such as BCA and pathogen inoculum concentrations following the initial treatment.

**Figure 1:**
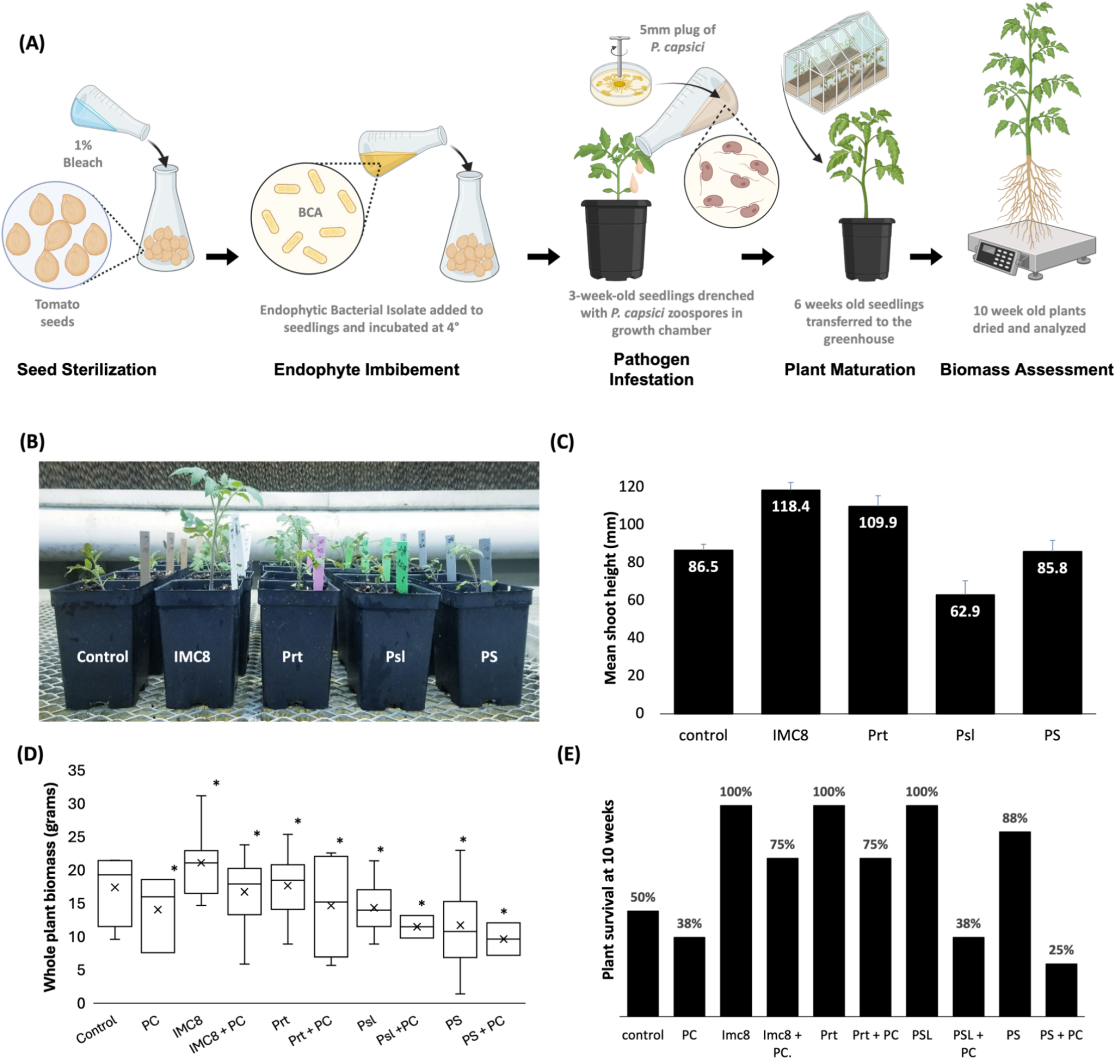
Efficacy of biological control agents (BCA) on tomato seedlings inoculated with *Phytophthora capsici*. (A) Schematic representation of tomato seed treatment with bacterial isolates. Tomato seeds were sterilized in 1% sodium hypochlorite (Clorox bleach) for 10 min, rinsed three times in sterile water, and blotted dry using heat-sterilized paper towels. Treated seeds were then soaked in BCA bacterial suspensions of 10^8^ colony forming units (CFU) for 24 h at 4 °C using *Bacillus thuringiensis* (IMC8), *Bacillus subtilis* (Prt), *Bacillus amyloliquefaciens* (Psl), and *Bacillus vallismortis* (PS). Groups consist of 10 to 12 individual plant replicates per treatment. The seeds were then sown in heat-sterilized soil in a growth chamber maintained at 26 ± 3 °C and were assessed for shoot height after 3 weeks (B, C). Plants were subsequently transplanted into nonsterile soil, inoculated with *P. capsici* using 10^5^ zoospores/mL, and transferred from the growth chamber to a greenhouse environment. Ten-week-old tomato seedlings were evaluated based on whole plant biomass (D), measured by plant dry weight, and plant survival rate (E), assessed based on the percentage of plants that survived from *P. capsici* root infections in a greenhouse. All error bars represent standard error . For whole plant biomass comparisons, mean values are projected with an x, while asterisks indicate significance (*t* test p-value of <0.05) for comparisons between each individual inoculation condition and the nontreated control.

**Figure 2:**
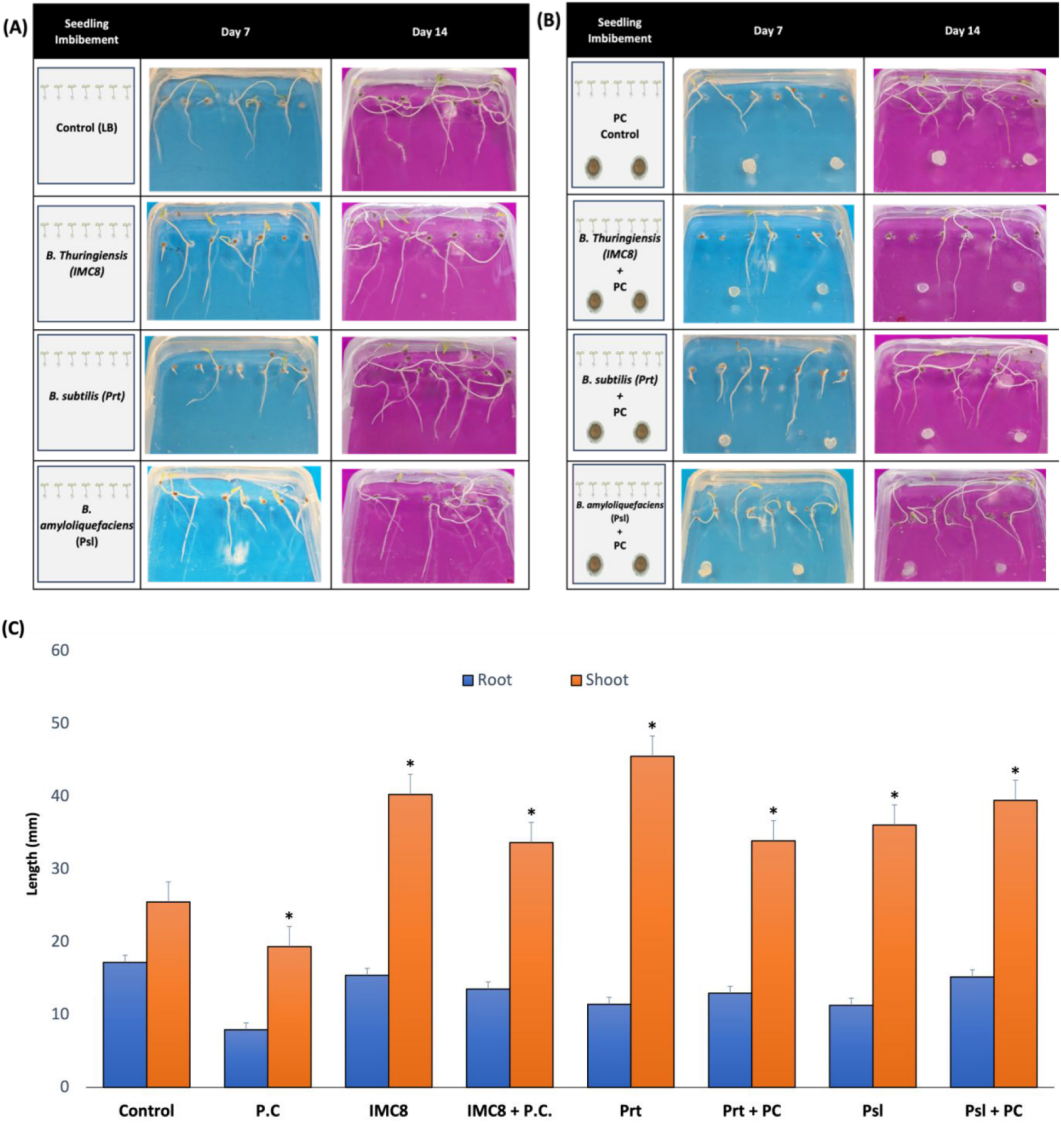
Alterations in the growth of tomato seedlings treated with biological control agents (BCA) and those with both BCA treatment and *Phytophthora capsici* (PC) exposure. (A) Groups where seeds were only treated with BCAs include applications of *Bacillus thuringiensis* (IMC8), *B. subtilis* (Prt), and *B. amyloliquefaciens* (Psl). Each group consist of seven individual plant replicates. (B) For pathogen exposed groups, 5 mm plugs of actively growing *P. capsici* were placed equidistance from the seeds, approximately 35 mm from the bottom of the plate. In both the BCA only and the pathogen exposed groups, blue and pink backgrounds represent day 7 and day 14 time points, respectively. (C) Mean root and shoot measurements were recorded on days 7 and 14 using ImageJ. Error bars represent standard error, with asterisks indicating significance (*t* test p-value of <0.05) for comparisons between individual inoculation conditions and their respective control. For these comparisons, seeds without any treatment acted as the control for BCA-only conditions, while seeds treated with the pathogen only represent the control for BCA and pathogen groups.

Tomato seedlings treated with BCAs and exposed to *P. capsici* exhibited longer roots and shoots after 14 days (Figures 2C). Shoot height increased significantly in plants treated with Prt (*B. subtilis*; 78.5% increase), IMC8 (*B. thuringiensis*; 57.9% increase), and Psl (*B. amyloliquefaciens* *;* 41.4%) compared to the nontreated controls. Seedlings exposed to *P. capsici* without BCA treatment displayed a 53.9% decrease in root length and a 24.1% decrease in shoot height compared to nontreated control. However, IMC8-treated plants exposed to *P. capsici* displayed only a 10.3% decrease in root length compared to nontreated controls. The more pronounced inhibition of root growth compared with shoot growth may be a consequence of pathogen inoculation via soil drenching, which exposes the roots to the pathogen prior to the shoots. With BCA exposure, there was an increase in root length observed in pathogen exposed seedlings treated with IMC8 (94.4%), Prt (63.3%), and Psl (91.6%) compared to those with only pathogen exposure. BCA-treated plants exposed to *P. capsici* pathogen also exhibited a significant increase in shoot heights compared to non-BCA-treated plants exposed to *P. capsici*, with increases of 74.0%, 75.2%, and 103.9% observed for IMC8, Prt, and Psl, respectively. These results suggest that biological control bacteria have a potent inhibitory effect on *P. capsici* growth and structural integrity, highlighting their potential as effective agents for managing this plant pathogen.

### Microscopy and DESI-MSI Analyses

Microscopic analysis revealed notable changes in the morphology of *P. capsici* hyphae within the zone of inhibition in response to the BCA after 48 h of cocultivation. Control *P. capsici* grown in the absence of BCAs exhibit hyphae that appeared healthy, with smooth, robust, and uniformly tubular structures (Figure S1). By contrast, as *P. capsici* hyphae grew closer to active culture of IMC8, they began to display signs of decreased structural integrity, including decreased hyphal branching (Figure 3A–D). Most prominently, there was widespread hyphal collapse within the zone of inhibition, characterized by the flattening and fragmentation of hyphae (Figure 3C–D). The affected hyphae experienced contraction, and their overall structural stability was reduced, suggesting disruption to the cell wall and membrane. Microscopic examination also showed that the hyphae near *B. thuringiensis* displayed cytoplasmic leakage and vacuolization (Figure 3D). These symptoms suggest that there is damage to the cells and a decrease in the internal pressure within the hyphal cells, causing them to collapse. These visual differences observed between the oomycete hyphae suggest that different metabolic states were induced within *P. capsici* when grown under *B. thuringiensis* exposure compared to the control. Variations in the assortment and quantity of small molecules present within a system can typically be correlated to the underlying biology, making the investigation of these small molecules integral for understanding any potentially altered metabolism.

**Figure 3:**
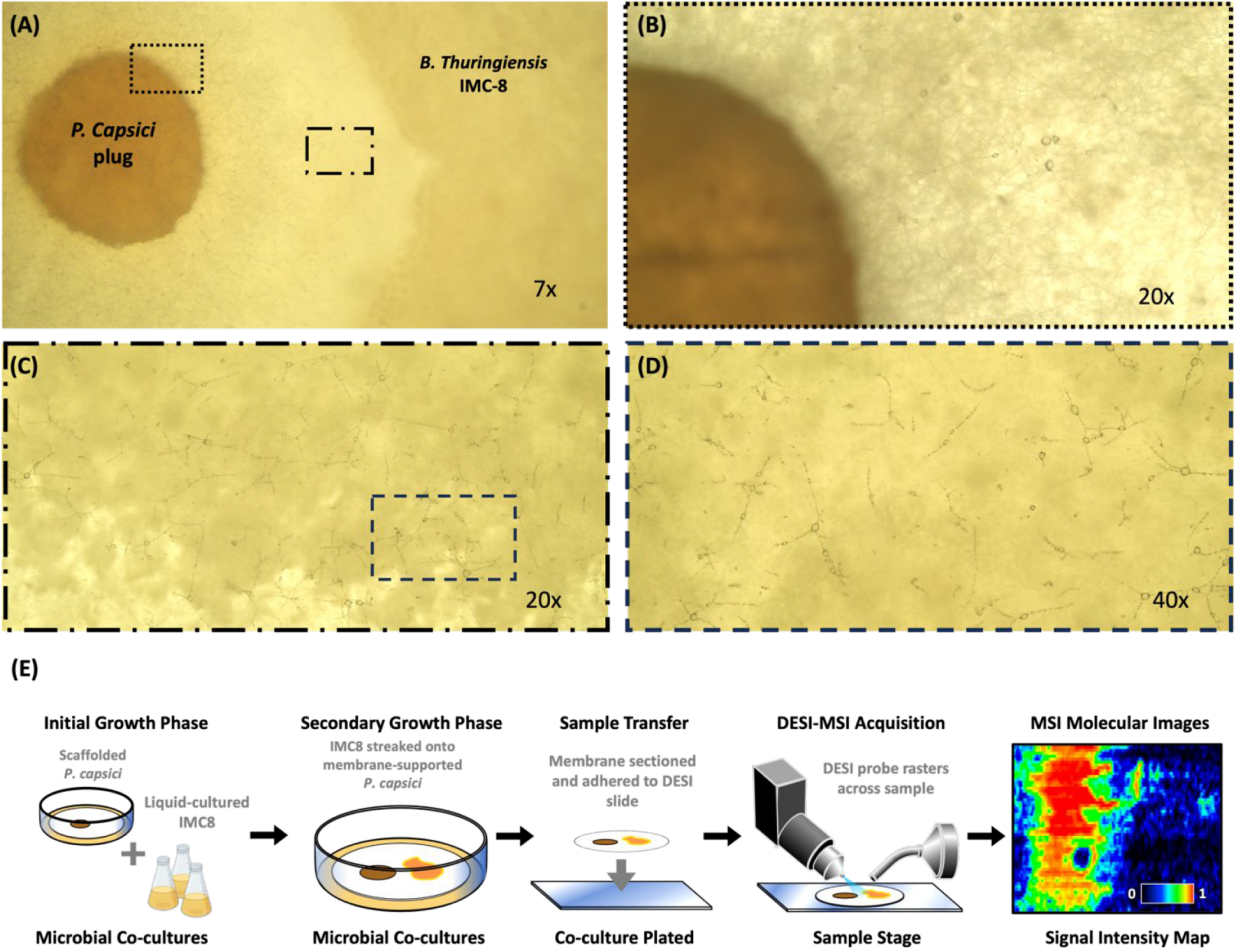
BCA effect on *P. capsici* hyphae morphology under optical microscopy. (A) Bright-field micrographs (7×) of a dual culture containing a 5 mm plug *P. capsici* growing concomitantly alongside endophyte, IMC8 (*B. thuringiensis*). (B) Normal hyphae morphology of *P. capsici* at 20×. (C, D) Zone of inhibition hyphae collapse at 20× and 40×. (E) Workflow diagram outlining the sample handling process for the *P. capsici* and *B. thuringiensis* coculture. Initial culturing of microbes occurs independently, prior to the microporous membrane scaffolding procedure. After cocultures are grown to the desired point, the membrane is removed from agar, cut to size, and affixed to a glass microscope slide for DESI-MSI acquisition.

To accurately characterize the spatial dynamics of the *B. thuringiensis* and *P. capsici* coculture, a DESI-MSI technique (Figure 3E) was implemented for the analysis of solid-phase bacterial growth cultured on a nylon membrane. By growing the coculture on a microporous membrane scaffold (MMS), active sample handling was minimized, allowing for the integrity of chemical signatures expressed during microbial growth to be preserved through the sample processing and analysis.^30^ The in situ molecular analysis achieved by the MMS technique combined with DESI-MSI was conducted under ambient conditions. Unlike vacuum-based MSI, the ambient nature of the DESI-MSI acquisition provides a better ability to measure labile and volatile analytes, which is integral for comprehensive characterization.^31^ Importantly, whereas prior work using MMS was only applied to cocultures of Gram-negative prokaryotes, this study represents the first MMS DESI-MSI investigation of an interdomain microbial coculture, which suggests the MMS approach is effective for diverse microbial samples.^31^ This particular experimental design emphasizes the versatility of the MMS platform by allowing for the coculturing of species with dynamic growth conditions and time scales. Here, we demonstrate the effectiveness of MMS and DESI-MSI for the metabolic probing and spatial characterization of BCA-pathogen interactions, as evidenced by the resulting spatially resolved metabolite identifications

The spatial distributions of 4000 total analyte features were measured for the bacteria-oomycete coculture during the DESI-MSI acquisition and processed into location-resolved chemical profiles (Figure 4B). In order to identify the spatially relevant and statistically significant features, complete chemical signature information was analyzed using an unsupervised segmentation algorithm.^26^ This spatially shrunken centroids (SSC)-based k-means clustering algorithm demarcated seven chemically distinct regions within the coculture, disregarding signals corresponding to noise and analytes with homogeneous spatial distributions that are characteristic of chemical background (Figure 4B). By comparing the output of the segmentation algorithm to the optical image of the sample taken prior to imaging (Figure 4A), these seven segments can be qualitatively assigned into three general regions. Among the chemically distinct regions determined, there were three segments associated with different phases of BCA growth (Segments 5, 6, 7), three segments of interspecies interaction (Segments 2, 3, 4), and one segment associated with oomycete growth (Segment 1, Figure 4D). The regions of interspecies interaction (Segments 2, 3, and 4), are particularly relevant for understanding the altered metabolic state arising in coculture conditions, as these are the areas in which the two species are in chemical contact with one another and correspond to the zone of inhibition identified by microscopy (Figure 3A).

**Figure 4:**
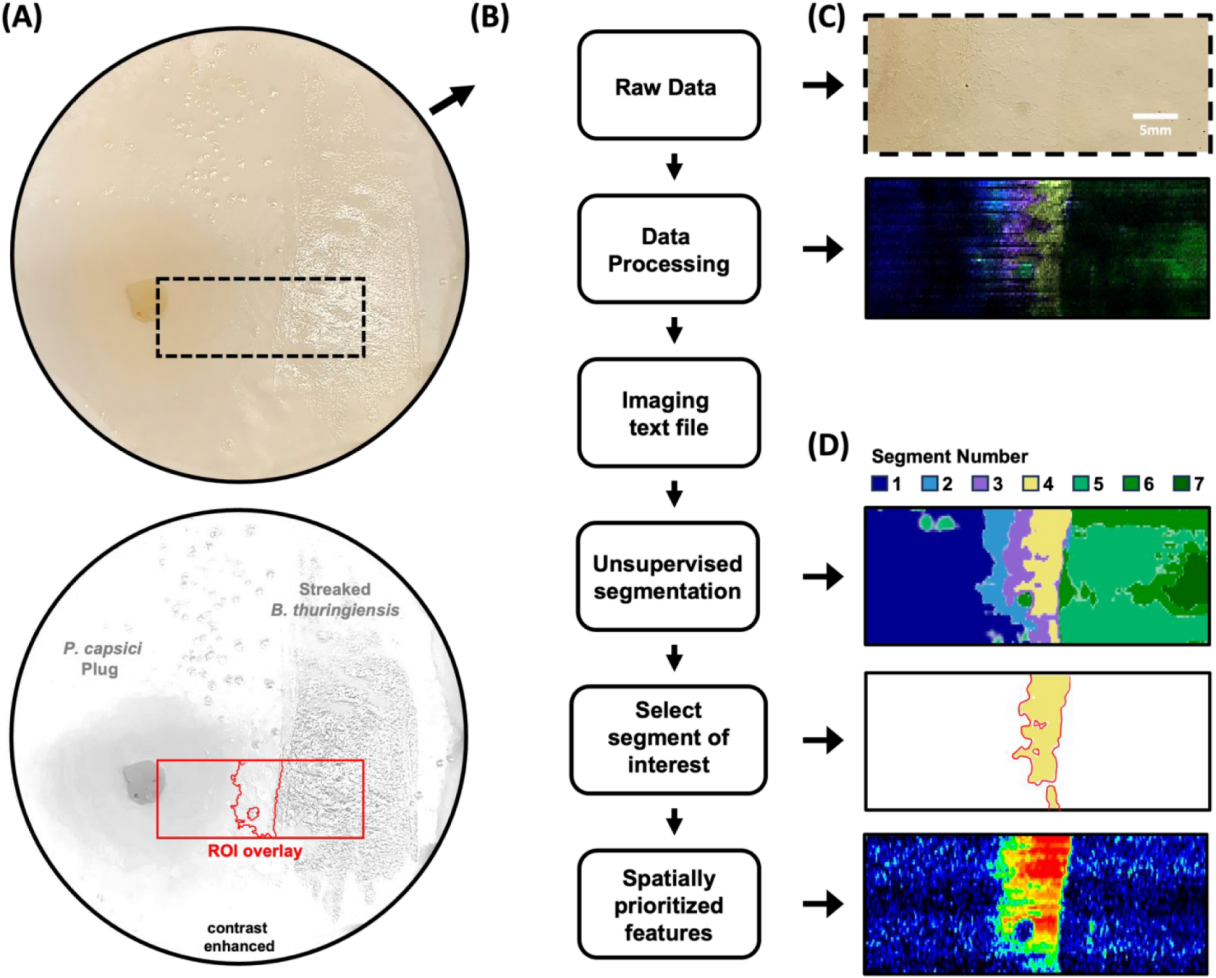
Segmentation workflow for spatiochemical profiling of *P. capsici* and *B. thuringiensis* coculture. (A) Optical image (top) of coculture grown on nylon membrane scaffold to preserve spatial location of chemical signals during sample transfer and data acquisition. Contrast enhanced image underscores the locations of the two species and the region of interest (ROI) being analyzed during DESI-MSI. (B) Data analysis workflow for application of the unsupervised segmentation algorithm. Raw data is processed in HDimaging before being imported into R, where the cardinal package performs an unsupervised segmentation algorithm using spatially shrunken centroids to determine regions with distinct chemical profiles. (C) Ion overlay displays selected ion images; different colors correspond to different molecular signals. (D) Output of spatial segmentation analysis, wherein 7 distinct spatiochemical regions were determined in an unbiased manner by the algorithm. Scale bars are as shown.

From the 4000 total analyte features measured from broadscale DESI-MSI analysis, 584 spatially relevant analyte signals associated with the metabolic states of both *B. thuringiensis* and *P. capsici* were identified via unsupervised segmentation. The location-resolved nature of these signals can be visualized in the form of ion overlays, wherein each of the seven segments occupies discrete regions within the imaged area (Figure 5A). Each of the 7 segments displayed unique chemical profiles, in terms of both the classes of analytes present as well as the intensities of the signals measured (Figure 5B). Feature significance is determined by the degree of contribution a signal has in the determination of individual spatial clusters within the SSC framework, with positive t-statistic values corresponding to greater contributions in distinguishing unique spatial segments as a result of abundance and feature. The prioritized (t-statistic >0) analyte signals present in Segment 1 (378 features) include lipids of the lysophosphatidylcholine (LysoPC) class as well as lysophosphatidylinositol (LysoPI) lipids, saturated free fatty acids (FFAs), and unsaturated FFAs. The significant features in Segment 2 (458 features) were predominantly nonlipid species, with the remainder being unsaturated FFAs. Five distinct classes of compounds were found to be significant in Segment 3 (130 features), with phosphatidylglycerol (PG) lipids, phosphatidylethanolamine (PE) lipids, saturated FFAs, hexosylceramide (HexCer) lipids, and nonlipid species being identified as significant in this region of interspecies interaction. Saturated FFAs, hydroxy-FFAs, PG lipids, and nonlipid species were the four classes observed to be significant in Segment 4 (47 features). Compounds from six classes were identified in Segment 5 (48 features). Ranked from the most number of significant features identified to the fewest, the classes are PG lipids, saturated FFAs, PE lipids, unsaturated FFAs, HexCer lipids, and nonlipid species. Segment 6 (37 features) is comprised of saturated FFAs, unsaturated FFAs, nonlipid species, and hydroxy-FFAs. The significant constituents of Segment 7 (58 features), occupying the region furthest from the oomycete-dominated region of Segment 1, were identified to be comprised of saturated FFAs, unsaturated FFAs, PG lipids, hydroxy-FFAs, and LysoPC lipids. A complete description of annotations and significance for features observed in each segment can be found in Supplementary Table S2.

**Figure 5:**
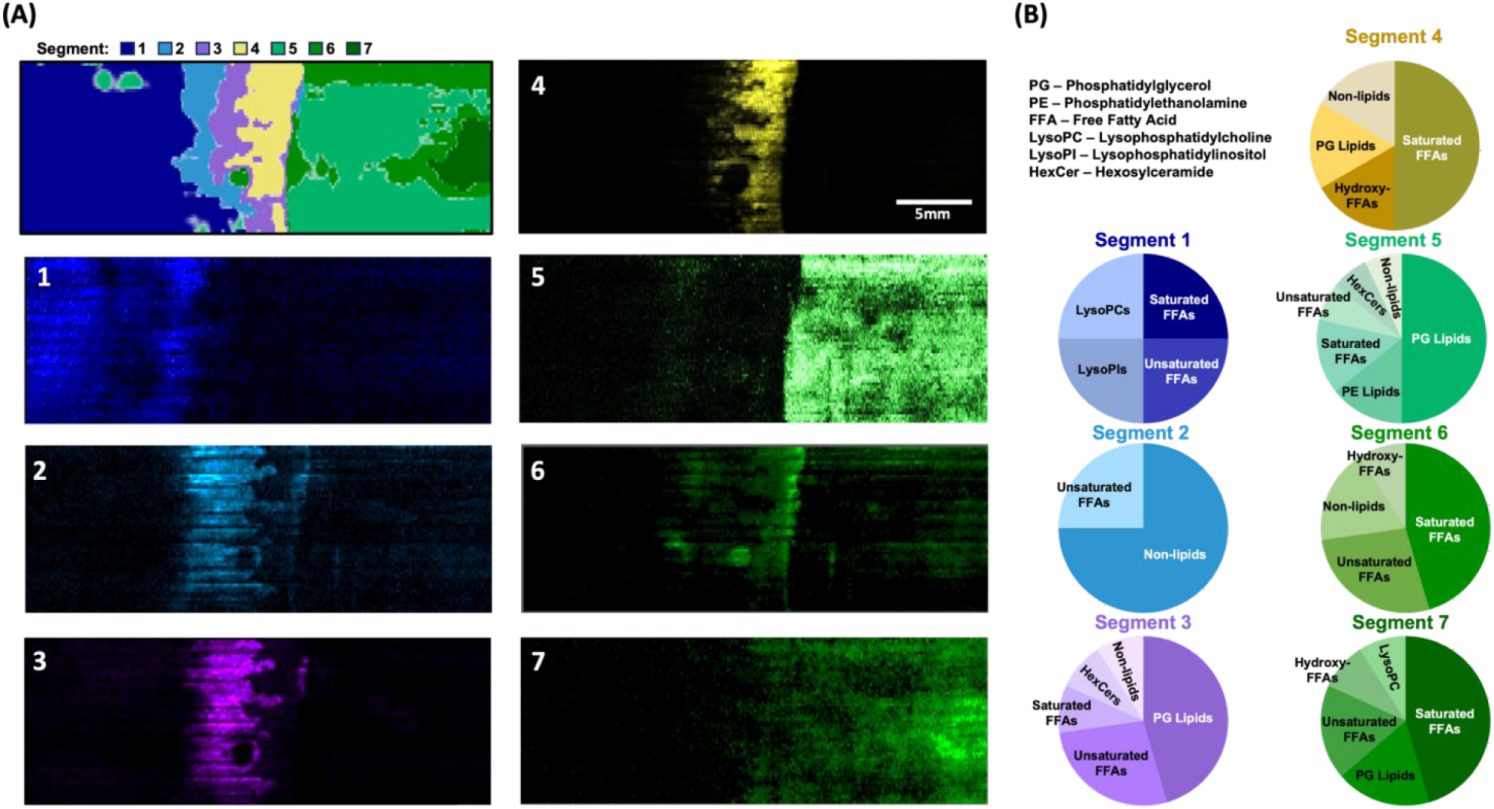
Breakdown of metabolic phenotypes identified by segmentation algorithm analysis of a DESI-imaged coculture of *P. capsici* and *B. thuringiensis*. (A) Output of segmentation algorithm (top, left) and ion overlays (1–7) for signals found in each of the seven regions determined by the SSC-based k-means clustering. Ion overlays serve to convey exact spatial localization of signals contributing to the segments identified by image analysis algorithm. (B) Class breakdown of identified features comprising the significant signals found within each of the phenomic regions unbiasedly determined through segmentation. Pie charts represent class breakdown for top 20 most significant features, with identifications of signals being determined though mass accuracy calculations using a 10 ppm threshold.

For the two distal regions of the image (Segments 1 and 7), a limited number of nonlipids were observed, with all of the significant analytes identified in these two segments being lipids. Based on the optical image overlay, these regions are associated with areas principally occupied by each the two individual species, *P. capsici* and *B. thuringiensis*, respectively. In these distal regions, FFAs dominate the observed signals in terms of measured abundances, with both saturated and unsaturated FFAs as well as both even and odd chain FFAs being represented. In contrast to these observations, the regions marked by interspecies interaction (Segments 2–4), have major significant signals associated with nonlipid species. Two of the nonlipid species found to be significant within Segment 2 include signals associated with hexose and heptose sugars. These sugars are identified primarily by accurate mass measurements, and thus represent numerous possible isomers which would necessitate additional experiments (e.g., chromatography, tandem MS, ion mobility) to inform the specific sugar isomers present. By comparing the chemical class distribution associated with each segment, information surrounding the implications of altered metabolic states brought about by plating these microbial species together can be discerned.

These broadscale chemical imaging results provide insight into the specific chemical ecology present in the coculture by identifying key metabolites contributing to the phenotypic profiles of each segment while simultaneously characterizing their spatial distributions. Most of the identifiable significant differences observed between the segments are due to variations in the lipid profiles, namely in terms of the lengths and the degrees of saturation of the lipid aliphatic chains, present in both the FFAs and lipid tails. For example, no significant signals identified as unsaturated FFAs were observed in Segment 4, while this class comprises a meaningful percentage of the significant signals present in every other segment. It is known that the degree of lipid saturation plays an important role in membrane fluidity, membrane permeability, and in cellular stress responses, with saturated fatty acids corresponding to more rigid and less permeable membranes that are less susceptible to oxidative stress due to the lack of double bonds along the carbon tail.^32^ The lack of significant unsaturated FFAs present within the interspecies interaction region (Segment 4) could correspond to a cellular response occurring in the zone of inhibition favoring membranes that have the aforementioned qualities due to proximity to the pathogen. Another notable difference between the distribution of FFAs within the seven segments is the significant presence of odd-chain FFAs in every region except Segments 2 and 5. The presence of odd-chain fatty acids is to be expected based on previous investigation of these strains, although the biological interpretation of this observation within the context of spatial analysis is as yet unclear.^33,34^

Spatial distributions of individual *m*/*z* values and different analytes can be visualized using heat maps which reveal analyte specific locations within the imaged area (Figure 6). One of the major lipid classes identified within the coculture by DESI-MSI were PG lipids. Twelve PG lipids representing six different chain lengths with three discrete degrees of unsaturation were found to localize in specific regions within the coculture. This heterogeneous lipid distribution implies varying functionality resulting from structural differences and serves to underscore the statistical observations calculated during image analysis. The saturated PG lipids PG 29:0, PG 30:0, and PG 31:0, as well as the unsaturated lipids PG 32:2, PG 33:2, and PG 34:2, were all primarily concentrated in the area of interspecies interaction located between the BCA and the oomycete (Segments 2, 3, and 4). By contrast, the saturated PG lipids (PG 32:0, PG 33:0 and PG 34:0) were all principally found within the three regions qualitatively associated with *B. thuringiensis* (Segments 5, 6, and 7), whereas the three monounsaturated lipids identified were codistributed in the regions associated with both the oomycete and the areas of interspecies interaction (Segments 1, 3, and 4). Additional representative heat maps for FFA lipids can be found in Figure S2. Without the spatiochemical information provided by the MMS DESI-MSI analysis, attributing individual signals to specific species or regions within the coculture would be challenging. The utility of the increased depth of analysis garnered through DESI-MSI is particularly emphasized in the investigation of metabolite classes known to be produced by both species present in the coculture, such as these PG lipids and FFAs.

**Figure 6:**
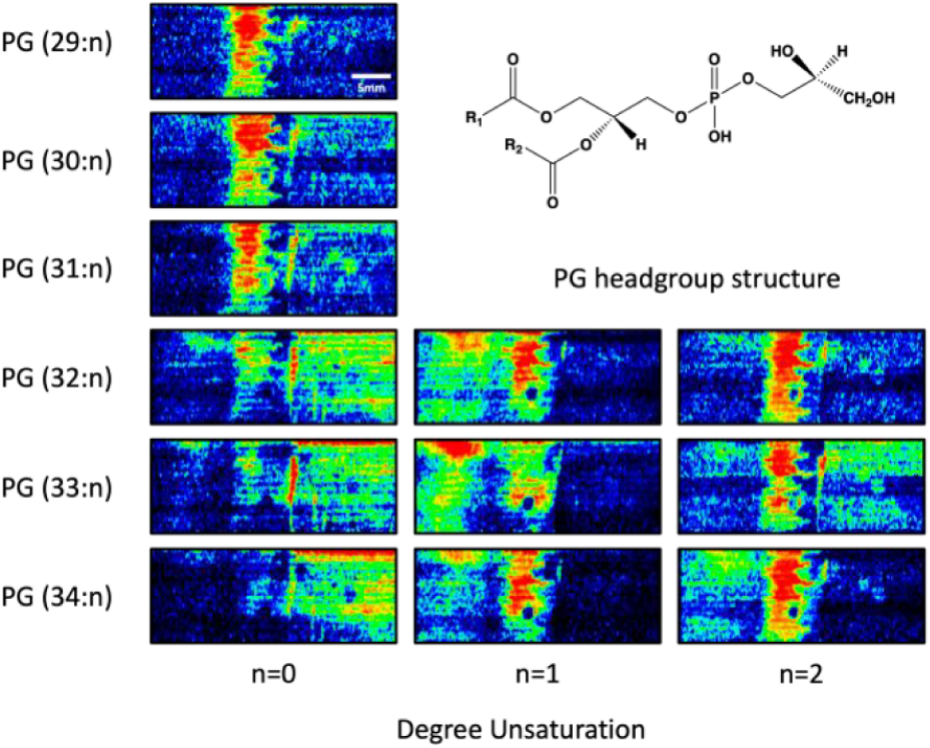
DESI molecular images of phosphatidylglycerols (PGs) reveal heterogeneous lipid distributions across the MSI analysis of a *P. capsici* and *B. thuringiensis* coculture. These MSI heat maps are organized by PG chain length (rows) and degree of unsaturation (columns), with chain length increasing from top to bottom, and sum number of double bonds increasing from left to right.

PG lipids are known to play an integral role in prokaryotic membrane function (e.g., charge-based self-assembly, protein translocation, initiation of replication, etc.) and there is literature precedent for the presence of PG lipids in the vegetative growth for both species studied.^33,34^ Furthermore, the PG lipid features tentatively identified in this study are consistent with previous ambient MSI and IM-MS investigations of *Bacillus* strains, including those specifically probing the lipidome of *B. thuringiensis*.^34–37^ Because these heat maps are spatially normalized, heterogeneous chemical signal distributions can be directly associated with physiological responses occurring in distinct regions of the microbial interaction. If these signals were simply a reflection of background lipid composition, they would be expected to be either uniform across the entire image or restricted to a single species of origin. Instead, the observation made from DESI-MSI analysis that specific lipid signals are uniquely concentrated within the area of interspecies interaction suggest that these molecules are either produced or accumulated in direct response to the oomycete-bacterial interaction rather than as general components of either organismal membrane. In particular, the observed increase in saturated lipids and FFAs within the interaction zone suggests a physiological adaptation to interspecies stress. Increased membrane rigidity, reduced permeability, and resistance to oxidative degradation are well-documented microbial responses to competitive and hostile environments. The localization of these lipid species within the interspecies interaction zone is therefore strong evidence that the interaction between the two species drives metabolic shifts in each organism’s lipidome. While the increased prevalence of certain PG lipid signals points to a physiological response, the FFA signals are likely markers associated with lipid degradation. This study marks the first instance where the analysis of living bacterial-oomycete cocultures provided observation of heterogeneous spatial distributions for PG lipids and FFAs, which is directly relate to a broader molecular understanding of the biochemical processes of microbial BCAs. Future investigations will seek to implement complementary orthogonal techniques as well as time-resolved acquisitions in an effort to expand the biological interpretability of the metabolic shifts observed in this present study.

## Conclusions

In this work, the effectiveness of *B. vallismortis*, *B. amyloliquefaciens*, *B subtilis*, and *B. thuringiensis* as growth promoting and biocontrol agents against *Phytophthora capsici* in tomato was evaluated. While *B. subtilis* and *B. thuringiensis* were observed to promote plant growth and provide pathogen protection against *P. capsici* in both in vitro and in growth chamber environments, *B. vallismortis* and *B. amyloliquefaciens* were found to be less effective in nonsterile soil greenhouse studies. The analysis of a pathogen-BCA coculture model via DESI-MSI highlighted the interactions and chemical signaling occurring between *P. capsici* and *B. thuringiensis* by investigating the molecular interactions that serve as the foundation for the underlying biochemistry. DESI-MSI was demonstrated to be an effective analytical strategy in the spatial analysis of the bioactive compounds generated by BCAs, providing deeper insight into the benefits of BCA application to tomato cultivation. While this work is presented here serves as proof of concept underscoring the analytical utility of direct ambient MS analysis to provide molecular insights into plant-microbe symbiosis, future work will focus on time-course studies exploring the fate of specific molecular markers, as well as incorporating ion mobility separation, which we believe is important for resolving isomers and supporting structural identifications, and for recovering peak capacity lost with the omission of chromatography.

## Supplementary Material





## Notes

BCA strain descriptions; optical images of normal, untreated *P. capsici* morphology; heat maps of additional lipids displaying heterogeneous distributions; DESI-MSI and segmentation outputs for biological replicates, and summary of all detected features with associated t-statistics (PDF)
Tabulated DESI-MSI dataset with segmentation-based intensity values (XLSX)

## ABBREVIATIONS

BCA: biocontrol agents
DESI-MSI: desorption electrospray ionization–mass spectrometry imaging
FFAs: free fatty acids
HexCer: hexosylceramide
IMC8: *B. thuringiensis*
ISR: induced systemic resistance
LysoPC: lysophosphatidylcholine
LysoPI: lysophosphatidylinositol
*m*/*z*: mass-to-charge ratio
MMS: microporous membrane scaffold
MS: mass spectrometry
MSI: mass spectrometry imaging
PE: phosphatidylethanolamine
PG: phosphatidylglycerol
PGPB: plant growth-promoting bacteria
Ps: *B. vallismortis*
Psl: *B. amyloliquefaciens*
SSC: spatial shrunken centroids
