# Supplementary material for: Desorption Electrospray Ionization–Mass Spectrometry Imaging Provides Spatiochemical Information on Potential Biocontrol Agents against Phytophthora capsici Infection in Tomato Plants
Source: J Am Soc Mass Spectrom. 2026 May 7;37(6):1465–75. doi: 10.1021/jasms.6c00067 (PMC13237771; doi:10.1021/jasms.6c00067)
Supplement: Supplementary file 1 [file js6c00067_si_001.pdf]

Supporting Information

Desorption Electrospray Ionization-Mass Spectrometry Imaging Provides Spatiochemical Information on Potential Biocontrol Agents Against *Phytophthora capsici* Infection in Tomato Plants

Jamille Y. Robinson,[1]† Hawkins S. Shepard,[2]† Daniel Ambachew,[1] Peter J. Eyegheleme,[1] Jody C. May,[2] Margaret T. Mmbaga,[1] and John A. McLean[2*]

1 Department of Agricultural Science and Engineering, Tennessee State University, Nashville, TN 37209

2 Department of Chemistry, Center for Innovative Technology, Vanderbilt University, Nashville, TN 37235

[*]Corresponding Author Email: john.a.mclean@vanderbilt.edu

SI Contents:

Table S1 – Endophytic biological control agents ……………..………....…….….……………..S2

Figure S1 – *P. capsici* morphology……………………………………………..…..….…….S3

Figure S2 – Additional heatmaps of interest …………………………………..….....……….. S4

Figure S3 – Segmentation output of biological replicates ……….…………………..………… S5

Table S2 – Summary of all features detected in the DESI-MSI analysis.…….…………….S6

Table S1: Endophytic biological control agents

Bacteria code	Identity	Source	Type	Reference
IMC8	*Bacillus thuringiensis*	Flowering Dogwood Branch	Endophyte	(Rotich et al., 2019)
Prt	*Bacillus subtilis*	Papaya root	Endophyte	(Joshua & Mmbaga, 2020)
PS	*Bacillus vallismortis*	Papaya Stem	Endophyte	(Joshua & Mmbaga, 2020)
Psl	*Bacillus amyloliquefaciens*	Papaya Stem leaf	Endophyte	(Joshua & Mmbaga, 2020)

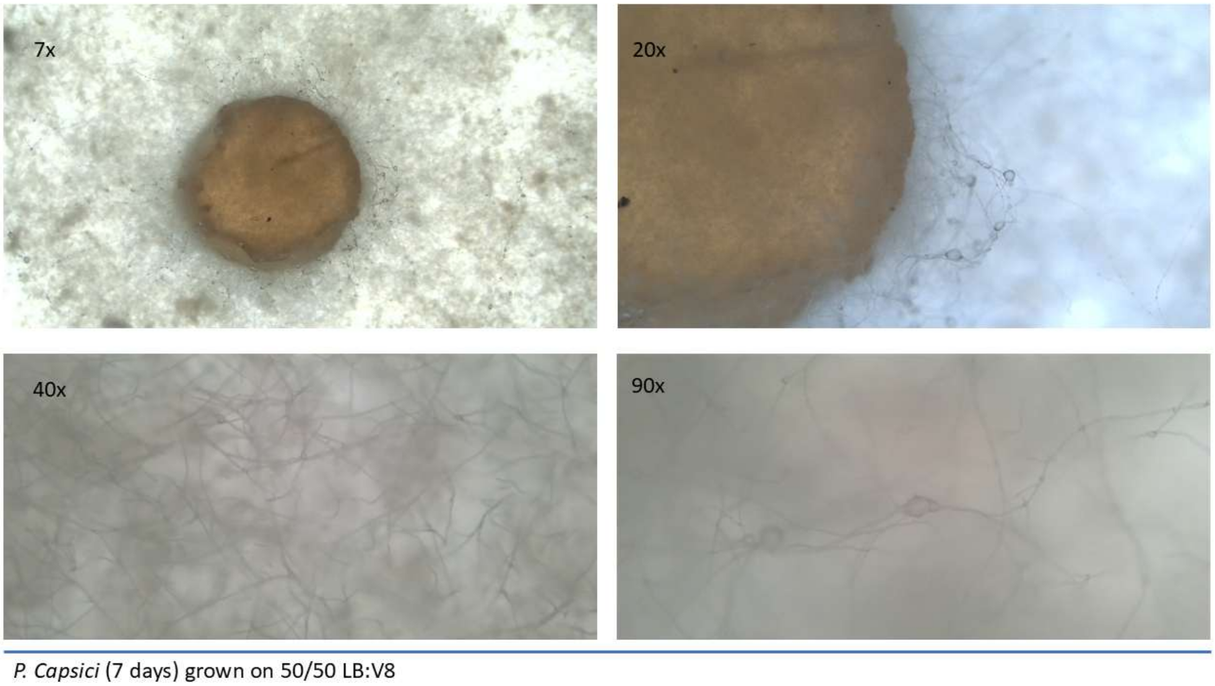

Figure S1: Normal *P. capsici* morphology on 50/50 mixed plates of LB and V8

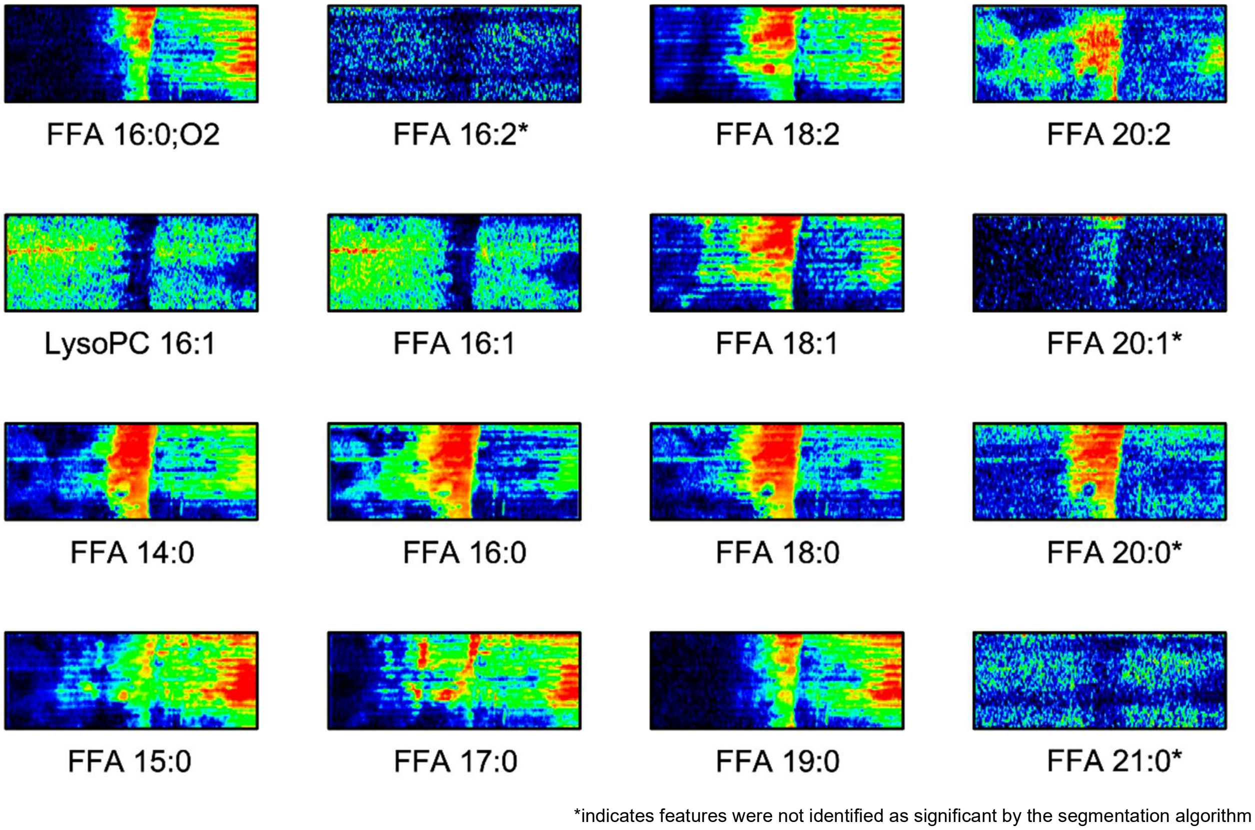

Figure S2: Additional DESI-MSI heatmaps for other lipids of interest.

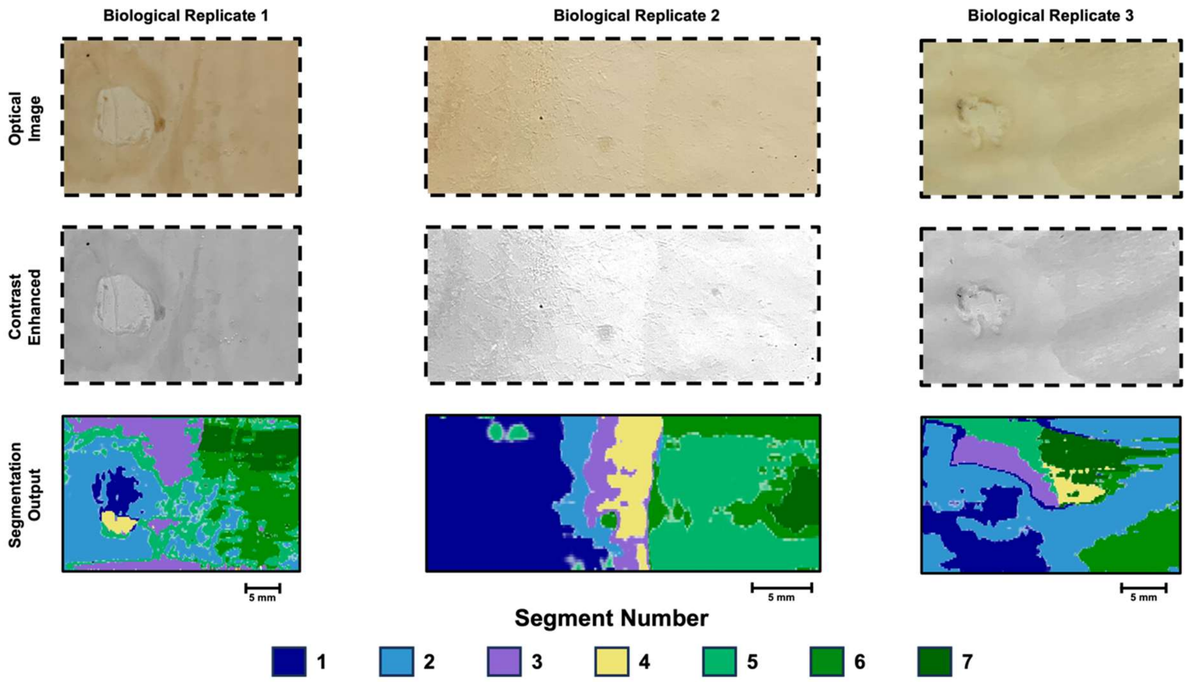

Figure S3: Segmentation output of biological replicates. All imaging data (optical images, contrast enhanced images, and segmentation outputs) represents interday acquisitions of three separate plates of dual-culture growth and subsequent DESI-MSI. To minimize process variability, culture conditions and plating orientations were kept uniform between replicates (5 mm *P. capsici* plug, 20 mL of 108 CFU / mL bacterial media streaked 3 cm to the right of plug). DESI-MSI segmentation results are shown for each individual replicate. Qualitatively similar segments identified across all interday acquisitions, with similar chemical profiles being present in each replicate, including spatially heterogenous phosphatidylglycerol (PG) distributions.

Table S2: Summary of All Features Detected in the DESI-MSI Analysis
(features for each segment can be found in the SI dataset)

m/z	Tentative Identification	Molecular Formula	Ion Form	Mass Acc. (ppm)	Total Abundance	T-Statistic						
						Seg1	Seg2	Seg3	Seg4	Seg5	Seg6	Seg7
50.8294					635053	1.2	0.0	0.0	-0.4	0.0	-1.4	-0.1
51.1102					549586	1.0	0.0	0.0	-0.6	0.0	-1.0	0.0
52.8088					579742	1.3	0.0	0.0	-0.1	0.0	-1.4	0.0
53.2339					550138	1.3	0.0	0.0	-0.2	0.0	-1.5	-0.2
53.7397					562427	0.5	0.0	0.0	0.0	0.0	-1.0	0.0
54.6347					535376	1.3	0.0	0.0	-0.9	0.0	-0.6	-0.4
55.4412					544422	0.8	0.0	0.0	-0.3	0.0	-1.0	0.0
55.6733					522988	1.5	0.0	0.0	0.0	0.0	-0.9	-0.2
57.0155					1896317	0.3	15.3	0.9	-10.9	-2.5	-13.4	-5.9
57.3341					542396	1.0	0.0	0.0	-0.1	0.0	-0.8	0.0
57.7895					522620	0.9	0.0	0.0	-0.3	0.0	-0.7	-0.1
59.0568					924777	2.6	0.0	0.0	-2.4	-0.2	-2.7	0.0
59.2769					524543	1.6	0.0	0.0	-0.7	0.0	-1.0	0.0
60.9757					2940893	17.6	14.4	-1.5	-17.1	-6.5	-21.6	-14.1
61.3549					491873	0.8	0.0	0.0	-0.3	0.0	-0.9	0.0
62.1311					4582758	24.8	12.6	-3.0	-20.5	-8.9	-25.3	-16.4
62.4189					475172	0.0	0.1	0.0	-0.2	0.0	-0.8	0.0
63.9734					519144	1.2	0.0	0.0	0.0	0.0	-1.2	0.0
66.3224					495135	1.2	0.0	0.0	-0.2	0.0	-0.9	0.0
66.9653					479192	0.9	0.0	0.0	-0.5	0.0	-0.7	0.0
68.5997					489855	0.9	0.0	0.0	-0.4	0.0	-1.2	0.0
69.6448					419621	0.6	0.0	0.0	0.0	0.0	-0.9	0.0
71.0081					679779	1.0	1.0	0.0	-2.2	-1.0	-2.2	0.0
71.5374					479338	0.8	0.0	0.0	-0.4	0.0	-0.5	0.0
71.5374					462913	1.1	0.0	0.0	0.0	0.0	-1.2	0.0
74.0644					460473	2.4	0.0	0.0	-0.7	-0.2	-1.8	0.0
74.5943					412419	0.9	0.0	0.0	0.0	0.0	-0.2	0.0
75.3012					423830	0.6	0.0	0.0	0.0	0.0	-0.6	0.0
76.1086					450896	0.4	0.0	0.0	-0.2	0.0	-0.7	0.0
77.0269					1955589	0.0	18.6	0.0	-10.7	-2.3	-13.5	-10.2
77.4566					403134	1.1	0.0	0.0	-0.2	0.0	-0.7	0.0
78.9913					616418	-0.1	1.6	1.6	-0.8	0.0	-1.9	-0.1
79.9775					450305	0.9	0.0	0.0	-0.1	0.0	-1.0	-0.2
81.0735					396982	0.7	0.0	0.0	0.0	0.0	0.0	0.0
81.5814					397792	0.6	0.0	0.0	0.0	0.0	-0.6	0.0
83.0386					1209220	0.0	9.9	0.0	-6.0	-0.2	-8.1	-3.4
84.1387					397197	0.4	0.0	0.0	0.0	0.0	-0.6	0.0
85.1191					819292	23.5	-15.8	-3.9	-4.5	-4.2	-5.4	4.7
86.0576					418868	1.7	0.0	0.0	-0.6	0.0	-0.8	0.0
87.0867					922418	-0.6	6.9	0.4	-5.1	-2.9	-5.1	0.4
88.1128					900761	12.4	-7.5	0.0	-6.2	-3.7	-6.2	8.0
89.0876					2520188	0.0	2.1	14.0	-12.6	0.0	-15.4	9.9
90.048					384259	0.4	0.0	0.0	0.0	0.0	-0.2	0.0
93.0267					5745403	-30.1	17.5	29.4	6.6	12.0	-9.6	-5.5
95.0409					7687406	16.7	37.5	-8.6	-28.6	-12.4	-34.1	-23.2
96.0943					765800	0.9	4.4	0.0	-3.0	-0.3	-4.1	-2.3
96.9609	Sulfate	H2O4S	[M-H]-	8.3	2442692	14.3	0.0	0.0	-13.6	-3.5	-16.3	4.7
97.9628					400130	0.8	0.0	0.0	0.0	0.0	-0.8	-0.2
99.1148					3075673	-13.3	35.3	4.6	-14.2	-0.5	-20.0	-11.0
100.2805					481631	0.0	1.5	0.0	-0.5	0.0	-1.1	0.0
101.0526					2544402	12.7	14.5	-2.9	-14.7	-5.4	-18.0	-11.4
102.8992					1589053	3.8	0.0	8.3	-10.5	-6.0	-11.1	8.7
103.1646					865851	14.5	-11.9	0.0	-4.7	-3.0	-5.4	9.1
105.1585					684356	9.7	-6.6	0.0	-3.7	-2.0	-3.7	5.2
106.0726					366188	0.7	0.0	0.0	-0.4	0.0	-0.5	0.0
107.0833					548328	0.4	1.1	0.0	-0.9	-0.1	-1.7	0.0
107.8964					328986	0.5	0.0	0.0	0.0	0.0	0.0	0.0
108.8948					1059115	-0.6	12.7	0.0	-7.1	-1.8	-8.5	-2.7
111.0747					1947403	-4.7	27.1	0.6	-16.0	-11.4	-14.5	-0.8
113.1604					822284	11.4	-9.8	-1.4	-5.0	-3.1	-4.9	11.4
114.0434					859014	20.5	-15.4	-1.7	-5.6	-4.6	-6.8	9.8
115.0495					781392	-3.5	12.0	0.0	-6.1	-3.7	-5.4	0.6
117.0497	Trimethylthiourea	C4H10N2S	[M-H]-	4.3	3646948	-38.2	56.9	8.3	-7.1	3.5	-13.0	-25.4
118.1773					671563	3.9	0.6	0.0	-4.3	-3.0	-4.3	0.1
119.034	2,3-Dihydroxybutanoic acid	C4H8O4	[M-H]-	-8.4	1366934	-6.0	15.7	0.9	-4.0	0.0	-7.3	-6.2
121.1665					1352796	0.0	7.3	0.0	-3.6	-0.9	-5.9	-2.7
123.1261					7017377	-3.5	48.8	3.0	-27.6	-5.5	-35.9	-20.2

123.9839					1429113	13.4	3.9	-1.3	-8.9	-3.9	-12.1	-6.5
125.0921					1145877	-2.6	-1.3	-0.3	3.6	0.0	10.0	-0.9
127.0451					1296736	2.6	8.4	-6.2	-11.7	-8.8	-11.0	11.5
128.1756					5306264	2.5	16.9	18.1	-25.5	-12.3	-29.0	8.1
129.042					1129658	-10.0	17.6	9.8	-6.1	-3.4	-7.5	-3.2
131.0688					5351681	86.3	-56.4	-15.4	-17.4	-15.2	-17.1	7.5
132.1639					2920304	42.9	-33.3	-4.6	-19.2	-13.4	-17.7	32.7
134.1886					1713676	32.5	-35.4	-9.7	-10.5	-9.6	-8.3	39.1
135.1651					3743631	10.6	0.8	3.6	-12.4	-4.3	-16.3	3.8
137.0224					6347388	0.0	43.2	0.2	-26.9	-6.0	-34.5	-19.2
138.0164					1221783	0.0	5.4	0.0	-1.8	-0.2	-3.7	-1.8
139.0033	2-Maleylacetate	C6H6O5	[M-H20-H]-	1.4	5762511	-15.6	52.4	5.6	-22.6	-3.6	-30.4	-18.6
143.1045					2221900	-16.9	30.5	2.7	-9.2	0.2	-14.2	-6.3
145.0941					5749155	13.2	13.4	-16.6	-25.0	-19.8	-21.9	23.4
146.0427					9384029	28.0	-4.5	10.9	-31.0	-18.9	-29.7	20.5
146.0427					2591544	-7.1	22.2	-1.1	-6.7	0.0	-9.8	-17.5
148.0456					821768	13.9	-9.1	0.1	-5.8	-4.0	-5.6	6.0
149.0434					870835	0.0	1.5	0.0	-0.4	-0.2	-1.2	0.0
151.1096					1052022	-19.5	25.2	12.2	-4.9	0.0	-5.0	-8.7
152.0377	2-Fluoroadenine	C5H4FN5	[M-H]-	-0.7	837888	11.5	-3.5	-2.0	-2.7	-2.9	-4.1	0.0
154.945					50477894	13.8	71.1	0.0	-49.5	-15.1	-61.7	-39.3
156.0604					5412818	8.3	37.1	-0.2	-26.2	-7.9	-33.2	-20.6
157.0846					7592545	16.8	24.6	0.1	-30.8	-13.3	-26.4	-13.5
158.0808	2-Pivalamidoacetic acid	C7H13NO3	[M-H]-	-9.5	1051612	9.2	2.0	-0.5	-6.4	-2.6	-6.3	-4.7
159.0748					1734401	52.7	-40.4	-9.1	-13.9	-11.1	-12.8	21.3
160.0596					1084589	35.2	-23.4	-7.5	-9.6	-7.8	-8.8	10.7
161.0914					639974	1.1	0.0	0.6	-2.5	-0.8	-3.3	0.8
162.9583					20567651	-7.7	71.9	5.9	-39.5	-8.2	-50.3	-31.5
164.0699					4204419	44.0	-43.0	-3.7	-14.4	-10.9	-14.9	38.4
168.1439					3306554	-15.0	10.7	32.8	-12.6	0.0	-17.7	8.4
171.1376	FA 10:0	C10H20O2	[M-H]-	-8.8	7018836	-43.7	29.1	4.6	17.9	9.5	-16.8	7.5
172.9566					26472741	27.6	53.6	-5.9	-43.3	-16.5	-54.8	-37.0
174.0544					2980313	3.8	30.6	-1.7	-21.3	-4.8	-21.1	-17.3
177.0362					8056498	-18.4	60.0	6.1	-26.1	-3.3	-35.4	-20.9
179.0555	Hexose Sugar	C6H12O6	[M-H20-H]-	-3.4	3447867	-3.8	33.1	0.0	-16.1	0.0	-21.5	-16.7
181.0713	Sugar Alcohol	C6H14O6	[M-H20-H]-	-2.8	1959257	5.6	-10.7	13.5	-3.9	0.0	-3.2	5.7
185.1538	FA 11:0	C11H22O2	[M-H]-	-4.9	2738047	-20.6	-0.8	-5.9	25.7	7.8	8.2	6.3
187.0973	1,9-Nonanedioic acid	C9H16O4	[M-H]-	-1.6	4312736	7.2	-16.8	-9.6	7.6	-2.6	25.9	0.0
188.9388					57442691	-3.2	81.0	4.0	-47.4	-10.3	-60.2	-36.4
190.0445					1573981	-0.7	13.8	0.0	-6.4	-0.3	-8.4	-6.7
191.0554	Heptose Sugar	C7H14O7	[M-H20-H]-	-1.0	3006154	-32.0	56.3	0.0	-15.5	-11.7	-13.0	-5.9
193.1589					604828	0.0	-2.2	1.7	0.0	0.4	0.0	0.6
195.0516					6209655	-12.9	53.8	5.7	-25.8	-3.0	-33.6	-21.0
196.0719					3628134	74.4	-57.3	-15.1	-19.7	-15.5	-17.5	30.8
199.1697	FA 12:0	C12H24O2	[M-H]-	-3.5	11276369	-32.9	-27.8	-3.4	44.6	11.4	25.2	39.4
202.1087					1776933	32.3	-31.7	-5.7	3.1	-4.9	2.6	3.2
204.9134					6235549	-5.3	47.1	5.0	-26.3	-1.6	-37.3	-19.5
206.9123					626121	0.0	5.0	0.0	-2.5	0.0	-3.7	-1.9
209.0775					1367680	0.0	-1.1	1.9	0.9	0.0	1.3	-1.5
213.1855	FA 13:0	C13H26O2	[M-H]-	-2.3	9370998	-27.4	-26.3	6.7	28.9	2.7	13.5	47.6
215.1241					3929205	-46.0	80.4	3.9	-19.2	-12.4	-16.1	-21.3
217.0430					3714456	0.0	24.5	-4.3	-10.4	0.0	-15.4	-16.5
223.0279					4074560	-1.0	32.3	2.4	-22.4	-2.9	-24.3	-10.7
224.9066					2704970	0.0	29.3	-7.4	-11.3	-0.1	-17.4	-18.7
227.2007	FA 14:0	C14H28O2	[M-H]-	-1.8	118143653	-34.5	-37.4	-8.8	57.3	8.0	100.6	-1.0
228.9319					37351859	7.1	72.0	4.3	-60.6	-13.6	-63.3	-25.9
232.9249					9225803	21.3	40.1	0.0	-44.5	-12.0	-43.7	-20.9
234.9232					1420529	0.0	15.1	0.1	-9.1	-0.8	-10.2	-9.1
238.9035					5641815	0.0	45.7	2.6	-27.3	-4.4	-36.1	-23.1
241.2174	FA 15:0	C15H30O2	[M-H]-	0.4	100061284	0.6	-45.8	-7.9	-5.9	-11.2	-3.8	101.5
242.2209					16757744	0.0	-42.3	-8.0	-5.7	-11.2	-5.0	98.1
244.9080					7321095	0.0	49.6	2.1	-26.2	-4.9	-36.6	-28.9
248.0800					4167073	-27.9	-28.4	-3.7	56.1	18.3	51.4	-7.7
250.1450					2197346	-6.5	14.1	-1.5	1.9	2.8	0.0	-15.0
253.2168	FA 16:1	C16H30O2	[M-H]-	-2.0	5572004	-25.0	-7.3	54.9	4.5	2.8	10.5	0.0
255.2324	FA 16:0	C16H32O2	[M-H]-	-2.4	276805785	-38.9	-37.2	-1.7	69.5	17.4	95.9	-13.3
256.2354					49692015	-37.9	-36.7	-1.7	65.5	17.6	95.8	-12.4
262.9198					2815254	-1.3	22.1	0.5	-12.9	-1.3	-16.3	-8.3
265.1479					7592636	-19.4	-33.3	-4.9	53.9	22.3	30.9	-4.0
269.2481	FA 17:0	C17H34O2	[M-H]-	-1.9	24954647	0.0	-60.4	29.4	6.4	-0.1	4.2	71.9
270.2516					4845956	6.4	-40.0	22.9	-9.5	-4.1	-9.6	59.6
271.2274	FA 16:0;O	C16H32O3	[M-H]-	-1.8	3657088	-26.1	-27.8	0.9	46.9	10.3	50.9	2.6
279.2325	FA 18:2	C18H32O2	[M-H]-	-1.8	75565538	-13.0	-66.1	9.9	20.3	17.4	42.4	64.2
283.2634	FA 18:0	C18H36O2	[M-H]-	-3.2	106570894	-37.5	-40.1	-3.7	71.6	29.0	86.5	-12.3
287.2227	FA 16:0;O2	C16H32O4	[M-H]-	-0.3	6808150	11.6	-61.3	0.8	22.0	0.0	16.4	50.6
294.9042					48322459	10.6	71.1	6.4	-44.7	-4.4	-49.5	-62.8

297.2799	FA 19:0	C19H38O2	[M-H]-	< 0.1	12466928	21.6	-60.0	-14.8	16.3	-8.8	5.5	64.9
298.9428					2428101	2.3	21.0	-2.2	-8.9	-1.6	-11.1	-17.8
301.2166	FA 20:5	C20H30O2	[M-H]-	-0.3	5634437	-44.0	58.6	18.9	-14.8	6.9	-13.6	-19.7
304.8753					2019847	4.7	14.5	-2.5	-7.1	-5.2	-9.5	-13.1
307.2631	FA 20:2	C20H35O2	[M-H]-	-2.0	2543201	20.3	-38.9	-4.4	-9.3	0.0	-11.2	54.8
311.1685	Undecylbenzene sulphonic acid	C17H28O3S	[M-H]-	-0.3	21640341	-21.5	-26.2	0.1	34.7	20.1	32.6	-14.5
316.8790					1513105	-1.5	13.7	0.2	-6.2	-1.0	-8.6	-5.6
322.8880					3385317	-3.9	40.5	6.3	-17.0	0.0	-22.6	-31.2
325.1839	Dodecylbenzene sulfonic Acid	C18H30O3S	[M-H]-	-1.2	25994265	-9.6	-10.1	-1.2	5.5	1.4	4.4	-2.8
326.1871					5832422	-8.8	-9.6	-0.9	4.9	2.3	5.7	-4.6
328.8912					3468751	3.2	38.9	1.9	-23.0	-2.5	-26.1	-30.2
330.8745					3022690	6.6	26.6	0.0	-15.8	-4.3	-19.5	-23.3
334.8966					33907223	9.7	65.2	2.4	-46.2	-13.4	-59.4	-32.0
339.1989					9368987	-17.8	-19.6	-1.4	22.3	10.1	20.8	-4.3
344.8675					6081290	2.9	32.7	0.1	-22.6	-3.5	-27.2	-17.2
347.8858					3297464	11.3	21.4	-1.6	-17.7	-7.2	-21.9	-14.3
350.8709					8967240	8.8	45.8	0.0	-33.9	-7.2	-41.8	-24.7
352.8635					2862287	9.9	21.7	-1.5	-18.4	-4.1	-21.6	-13.5
354.8585					1126664	1.3	7.1	0.0	-5.0	0.0	-6.3	-3.9
362.8819					1433342	0.0	8.8	1.3	-4.2	-0.9	-6.1	-7.1
367.8823					2461242	13.0	14.8	-1.2	-18.4	-6.4	-16.7	-9.5
373.5728					2787578	2.8	19.2	0.0	-12.7	-3.0	-16.2	-11.9
375.8674					1707268	6.5	10.5	0.0	-9.6	-3.3	-12.3	-7.8
379.8721					1575979	11.3	3.6	-2.3	-7.2	-2.8	-8.5	-6.3
383.8577					2682829	1.8	15.8	0.0	-6.1	-1.1	-9.0	-15.8
386.8509					1411064	3.2	10.0	0.0	-7.9	-2.9	-9.7	-5.0
390.5667					1324598	0.4	9.4	0.5	-6.1	-2.0	-8.6	-4.1
393.5691					6972856	8.4	36.1	0.0	-26.7	-7.0	-33.5	-20.2
400.8674					26095584	20.2	54.2	-3.6	-42.9	-15.7	-53.0	-30.8
404.8368					1606319	1.5	11.8	0.0	-7.6	-1.5	-8.7	-7.5
410.8387					2582419	4.5	21.3	0.0	-14.9	-4.9	-18.0	-12.3
416.8413					5125597	12.0	34.1	0.0	-27.8	-7.5	-33.6	-20.2
418.8365					944516	0.0	7.2	0.0	-4.9	-0.3	-5.2	-2.7
421.2245					4738719	-33.8	-27.9	0.0	51.8	31.9	40.5	4.4
422.8434					1508002	-2.7	22.2	2.4	-10.0	-4.5	-9.9	-12.9
426.5544					13833119	14.8	49.0	-0.7	-37.3	-11.3	-44.4	-30.3
428.851					3008398	9.7	16.6	-1.1	-14.8	-3.8	-18.1	-12.5
431.5402					1434962	3.6	8.3	0.0	-7.9	-1.8	-8.3	-4.5
434.8544					4593958	-17.1	43.3	7.1	-12.5	0.9	-16.0	-26.9
437.2759					3914396	49.4	-55.8	-15.6	-8.6	-13.7	-10.1	53.1
440.8597					9242515	17.6	38.5	0.0	-30.9	-10.6	-38.2	-28.7
446.551					11298539	19.7	43.7	-0.9	-41.9	-12.1	-47.2	-22.4
448.5407					1084028	2.0	10.1	0.0	-8.1	-2.6	-8.6	-4.8
450.8309					4561639	6.0	20.1	0.0	-16.2	-3.7	-21.5	-11.1
454.5385					3924749	7.5	23.2	0.0	-19.3	-5.5	-23.4	-11.7
456.8359					4820939	12.1	27.9	-1.4	-22.6	-7.9	-27.8	-17.9
459.5355					1913462	3.6	11.4	0.0	-8.8	-1.8	-11.2	-7.2
462.5257					1501101	1.6	8.3	0.0	-7.3	0.0	-9.0	-3.1
464.5339					953697	3.0	5.7	0.0	-6.3	-1.8	-6.7	-2.7
468.5463					1815641	6.7	9.7	-1.1	-7.8	-2.1	-10.6	-8.4
470.5282					2084246	3.9	13.5	0.0	-9.9	-2.3	-11.9	-8.9
472.812					1261246	2.4	9.9	0.3	-8.4	-1.2	-10.4	-5.3
476.5331					883372	2.5	6.5	0.0	-4.1	-1.8	-5.9	-4.7
479.5366					11290564	16.6	41.4	-1.0	-34.8	-11.5	-41.6	-23.9
482.5376					3056146	6.6	22.1	0.0	-13.7	-3.1	-18.0	-20.2
485.5397					1134635	2.4	7.5	0.0	-5.4	-0.5	-7.6	-5.0
487.5239					2787757	3.1	21.6	0.1	-12.2	-1.4	-14.8	-19.2
488.8152					1605874	-1.1	16.6	0.3	-5.1	-0.3	-7.9	-13.1
493.5282					3516809	9.0	21.9	-0.4	-18.2	-5.8	-21.3	-14.1
496.5298					1585929	2.0	7.9	0.0	-6.3	-1.7	-7.7	-3.9
497.5223					1395270	7.2	5.9	-1.3	-6.8	-3.2	-8.5	-5.1
499.5323					5386283	8.9	26.8	-1.0	-23.1	-6.6	-27.0	-12.0
501.5213					1440442	4.7	10.4	0.0	-7.8	-4.3	-10.4	-6.5
504.5181					1654405	2.5	11.7	0.0	-10.0	-1.7	-12.9	-4.6
506.831					5763287	17.8	29.0	-1.2	-27.5	-9.9	-33.2	-19.3
510.5359					1568950	6.5	7.8	-0.8	-7.0	-2.5	-8.9	-7.8
512.5193					2299943	4.7	12.3	0.0	-10.0	-2.9	-12.2	-8.0
516.8019					1526609	3.2	12.0	0.0	-9.2	-2.4	-11.1	-6.9
518.5219					724348	2.7	3.9	-0.2	-3.0	-1.0	-4.7	-3.2
521.5281					4602396	13.3	23.2	-0.8	-19.2	-6.4	-24.6	-18.6
524.5277					2304160	8.3	16.1	-1.1	-12.3	-4.8	-15.7	-12.4
526.7762					784670	0.0	7.2	0.0	-3.9	0.0	-4.3	-4.2
528.2821	LysoPC 16:1	C24H48NO7P	[M+Cl]-	-7.8	2150776	30.5	-41.0	-11.5	-9.5	-10.7	-9.0	53.7
529.5151					1738094	4.8	14.6	0.0	-8.3	-2.0	-11.8	-14.8

532.5180					5190231	11.6	27.8	-0.5	-23.6	-6.7	-32.4	-14.1
535.5193					2778736	6.9	11.7	0.0	-11.0	-3.3	-12.7	-8.6
540.8200					1844117	3.3	11.5	0.0	-9.5	-2.2	-11.0	-6.7
543.5082					902094	1.5	6.0	0.0	-4.5	-1.0	-5.8	-2.7
546.8237					2534767	7.2	11.3	0.0	-11.5	-2.7	-14.4	-6.8
550.7922					1220796	-2.5	12.8	1.6	-4.5	0.0	-5.9	-7.9
552.5155					2664314	1.7	16.1	0.1	-13.9	-1.6	-14.8	-6.1
554.2618					202748494	-13.7	40.4	3.9	-5.4	-11.3	-47.6	4.9
555.2644					69829833	-5.7	78.3	8.0	-46.8	-13.8	-59.2	-29.2
556.2675					13854143	-11.6	30.6	2.6	-3.9	-8.6	-28.9	0.1
560.5031					939056	1.6	6.1	0.0	-5.9	-0.6	-6.7	-1.7
563.5190					3605630	13.5	13.2	-3.0	-11.8	-4.5	-15.6	-15.3
566.5184					2096217	7.7	13.3	-0.3	-11.5	-4.0	-13.8	-10.4
568.4988					502904	0.0	1.2	-0.1	0.0	0.0	0.0	-1.3
571.5048					1558157	5.8	4.8	0.0	-5.1	-2.3	-7.8	-5.2
572.7740					1269732	3.5	7.9	0.0	-6.4	-1.3	-8.3	-5.7
574.5093					3100962	4.4	25.7	0.0	-12.2	-3.1	-16.2	-25.0
576.2438					67479800	-8.5	39.0	-0.7	-5.1	-11.8	-44.1	0.6
577.2465					23695670	-0.5	66.3	3.8	-40.2	-10.9	-52.1	-27.6
578.2497					4582884	-4.8	35.2	5.5	-21.5	-4.4	-27.7	-9.9
582.4971					1436189	3.9	11.2	-0.8	-8.1	-2.6	-9.2	-7.0
583.5057					740571	2.5	2.4	0.0	-2.7	-0.3	-4.0	-2.4
585.4999					2471095	0.9	6.3	0.0	-4.2	-1.9	-3.1	-4.0
588.5003					1633064	4.2	9.5	0.0	-8.8	-3.1	-9.8	-4.7
592.2183					2325908	2.4	19.6	4.1	-22.2	-5.7	-19.6	-2.1
593.1272					4492421	-26.3	-27.1	-5.2	55.5	3.7	74.9	-11.1
594.7801					2254271	10.6	18.1	-0.9	-22.0	-4.3	-20.5	-8.5
596.4965					990448	1.5	7.6	-1.2	-4.5	-0.4	-4.6	-5.3
598.2255					52149349	3.4	72.8	0.0	-48.4	-12.7	-58.0	-32.6
599.2284					17391014	1.8	61.6	2.0	-38.3	-9.6	-48.8	-30.7
600.2315		C32H39NO8	[M+Cl]-	-9.2	3476919	0.0	32.0	0.8	-20.7	-3.8	-25.6	-13.0
601.2349					586936	0.0	1.6	0.0	-1.0	0.0	-2.3	0.0
602.7898					641880	0.0	3.7	0.0	-2.4	-0.2	-3.1	-0.5
605.5055	WE 39:4	C39H70O2		-2.5	1359453	3.3	7.3	0.0	-6.6	-1.4	-8.8	-3.4
607.4914					1016483	2.1	5.1	-0.1	-5.0	-0.6	-5.0	-2.4
609.2170					1622420	1.7	10.6	0.1	-8.1	-1.9	-10.4	-5.2
611.2833					3128197	1.8	14.3	-0.8	-10.1	-4.0	-14.9	-2.1
612.7952					3606292	11.3	24.0	-1.7	-20.9	-5.7	-24.0	-15.7
614.2034					3466924	-0.1	27.4	1.0	-14.5	-1.6	-21.8	-12.8
615.2059					1077784	-1.3	5.5	2.7	-3.3	0.0	-5.8	-0.1
616.5005					3014902	12.6	15.6	-1.6	-14.6	-4.8	-18.0	-14.4
620.2078					39310604	6.8	69.0	0.5	-47.4	-12.0	-56.7	-34.3
621.2111					12388868	5.3	51.1	4.3	-35.7	-10.3	-46.4	-24.2
622.2159					2394367	0.0	22.3	3.2	-14.4	-2.0	-18.0	-10.0
624.4881	Cer 36:3;O6	C36H67NO7	[M-H]-	5.8	1311944	2.5	10.5	-0.2	-5.9	-1.6	-8.2	-7.3
627.4901					2087209	4.5	12.7	0.7	-11.3	-1.5	-12.8	-9.0
630.4919	HexCer 29:0;O2	C35H69NO8	[M-H]-	-4.9	1663178	5.3	9.7	0.0	-8.2	-2.7	-9.8	-7.4
634.2030					1423727	-1.3	11.0	3.0	-2.7	0.0	-6.9	-9.5
636.1840					2887256	-0.3	22.3	4.0	-15.8	-2.4	-21.3	-5.8
637.1840					1147966	0.0	7.5	0.0	-4.3	-0.5	-6.4	-3.4
639.2406					1384700	2.0	6.6	-1.1	-5.6	-0.9	-7.2	-1.4
640.7789					1377587	7.6	11.0	-1.1	-11.8	-2.6	-12.4	-7.8
642.1898					18736751	3.2	47.3	6.6	-37.2	-8.0	-45.7	-17.1
643.1924					6414395	10.1	40.5	0.0	-28.7	-7.4	-36.6	-25.4
644.2028					1310607	1.9	11.6	0.0	-8.5	-1.5	-10.0	-6.1
647.4978	LysoPA 33:0	C36H73O7P	[M-H]-	-6.6	1082371	6.5	4.6	-1.9	-4.6	-2.2	-5.9	-5.6
651.2084					3235912	5.0	20.0	0.0	-13.2	-4.0	-16.7	-14.7
651.7100					2165220	2.4	16.6	0.3	-10.1	-1.5	-12.8	-13.3
652.7905					1259061	1.4	8.8	0.0	-5.4	-0.5	-7.7	-5.9
652.7905					869238	3.2	6.4	-0.5	-5.8	-2.2	-6.1	-3.7
658.1676					2006845	0.7	14.8	0.0	-9.7	-0.8	-11.6	-7.5
662.2004					5906630	6.0	20.4	1.1	-17.4	-4.8	-22.0	-10.2
664.1714					2166809	0.0	18.6	0.0	-13.7	-4.3	-15.5	-3.6
665.1757					775317	1.6	6.9	-2.3	-4.2	-4.1	-4.2	-2.0
666.4731					784015	0.0	3.3	1.9	-1.0	1.7	-3.0	-5.8
669.3988	LysoPI 23:0	C32H63O12P	[M-H]-	0.6	3057324	28.6	-39.8	-11.9	-12.4	-10.6	-10.3	58.3
670.1860					1476929	-1.1	11.0	0.5	0.0	0.0	-3.6	-11.4
673.1893					4562061	13.7	19.4	-3.9	-16.3	-5.7	-20.0	-16.8
676.2044					620342	0.0	4.9	0.0	-2.1	-0.1	-2.8	-1.2
678.1682					979828	0.2	4.5	0.1	-4.8	-1.2	-5.8	0.0
679.4550	PG 29:0	C35H69O10P	[M-H]-	-0.1	1574058	-16.6	-12.0	37.1	0.0	46.6	-3.1	-7.5
682.2024					1703686	0.0	10.9	0.1	-6.1	-0.8	-9.5	-3.2
684.1834					2269172	3.9	12.6	0.0	-11.0	-5.2	-12.4	-4.5
686.4731	PE 32:2	C37H70NO8P	[M-H]-	-5.1	1446857	-18.5	-12.7	6.9	20.0	55.7	0.2	-8.6
689.4820	PG O-31:2	C37H71O9P	[M-H]-	8.3	882366	-1.9	6.4	0.0	-1.2	2.8	-2.5	-5.0
691.1805					748120	4.1	4.6	-2.4	-3.7	-2.9	-4.2	-3.1

692.1720					733307	0.0	3.1	0.1	-3.0	-0.8	-3.2	0.0
693.4669	PG 30:0	C36H71O10P	[M-H]-	-5.3	1823423	-18.2	-15.5	35.0	7.6	44.4	-0.3	-5.0
694.4687					1000000	-10.7	-8.2	22.4	3.7	23.6	0.0	-2.7
695.1705					1601134	1.9	17.1	-1.0	-9.5	-7.0	-12.1	-7.8
697.2908					1154117	6.3	4.9	-1.1	-6.0	-3.1	-6.8	-4.2
698.2927					605771	2.2	0.8	-0.4	-1.5	-0.5	-1.6	-0.9
700.4839					1083637	-4.6	0.9	5.8	0.0	15.4	-5.2	-5.5
702.1935					511348	0.0	0.4	0.1	-1.8	0.0	-1.9	0.2
704.1910					10469276	12.9	40.3	-3.1	-28.6	-9.1	-37.0	-25.6
705.1928					4307813	15.7	27.9	-5.8	-22.4	-13.4	-26.0	-18.2
706.1939					942122	1.7	4.7	-0.7	-3.5	-4.0	-5.1	0.0
707.4819	PG 31:0	C37H73O10P	[M-H]-	-7.1	1530297	-12.8	-17.3	33.7	2.2	40.9	-3.5	-0.5
711.4727	PA 34:0	C37H73O8P	[M+Cl]-	-1.4	850097	0.0	2.7	0.7	-3.1	-0.3	-3.6	0.0
713.4733	3,4-Dehydrorhodopin glucoside	C46H66O6	[M-H]-	-7.6	4681421	14.0	-42.7	-1.6	33.6	5.2	15.5	4.3
715.1819					2966725	3.4	21.9	0.0	-16.1	-8.7	-14.8	-11.1
715.4718	PG O-30:0	C36H73O9P	[M+Cl]-	4.5	3982475	-23.0	-18.5	9.7	22.3	74.0	2.5	-10.4
718.1789					1904343	5.0	15.2	-1.2	-13.3	-13.9	-12.5	-0.9
719.4798	PG 32:1	C38H73O10P	[M-H]-	-9.9	2489791	-31.8	7.9	6.2	15.6	48.8	1.1	-15.2
721.4973	PG 32:0	C38H75O10P	[M-H]-	-7.2	2146511	15.3	-32.1	6.0	1.9	5.4	-5.9	22.0
723.1704					918448	2.5	4.6	0.0	-4.9	-1.1	-5.6	-2.4
726.1729					15217047	7.5	44.7	0.4	-34.6	-10.3	-43.1	-17.2
727.1753					5483320	10.2	32.5	-2.2	-23.9	-7.8	-30.1	-18.9
728.1779					1231892	2.6	12.1	0.0	-8.0	-3.8	-9.4	-6.9
729.1767					521869	0.0	3.1	0.0	-1.9	-1.4	-2.2	0.0
731.4844	PG 33:2	C39H73O10P	[M-H]-	-3.4	2147798	-8.1	-17.0	20.1	5.0	47.5	-5.7	-3.5
733.4974	PG 33:1	C39H75O10P	[M-H]-	-7.0	1741843	-25.2	19.4	0.0	10.0	12.9	2.8	-11.0
734.4956	PE O-34:3	C39H74NO7P	[M+Cl]-	8.0	973404	-14.5	12.4	0.0	5.0	7.4	1.3	-7.4
735.5113	PG 33:0	C39H77O10P	[M-H]-	-9.4	1332981	6.8	-21.8	-0.6	6.2	1.7	-2.0	20.5
737.1641					1033724	4.7	7.5	-1.3	-5.9	-1.7	-6.5	-7.2
740.1600					1414397	0.6	11.3	1.9	-10.0	-2.5	-11.6	-2.6
742.1483					2084380	1.4	15.4	-2.2	-10.3	-12.7	-11.0	0.0
743.1532					875327	2.7	5.1	-2.4	-2.2	-4.6	-2.2	-3.3
745.4983	PG 34:2	C40H75O10P	[M-H]-	-5.6	4166670	-27.2	-13.5	11.8	21.6	75.9	0.3	-12.1
746.5022	HexCer 34:3;O3	C40H73NO9	[M+Cl]-	5.8	1801630	-22.3	-13.1	11.5	17.3	65.0	0.7	-9.8
748.1548					4076038	4.5	24.1	3.4	-22.5	-14.7	-22.0	-4.0
749.1642					1786849	2.5	22.6	0.0	-12.1	-6.5	-11.7	-18.0
754.1722					657329	1.4	4.6	0.0	-2.6	-1.0	-3.8	-3.4
757.1728					1794511	9.1	11.8	-2.1	-9.4	-7.8	-11.1	-9.9
760.1715					1435197	4.6	11.0	-0.8	-8.1	-2.8	-8.4	-9.0
760.6756					1063966	5.3	8.5	-1.8	-6.8	-2.0	-7.6	-7.5
762.1453					1433928	6.0	11.5	-0.8	-9.2	-2.1	-11.2	-9.0
763.4597	PG 36:7	C42H69O10P	[M-H]-	5.4	1361451	15.8	-27.1	-7.7	-0.4	-6.7	-5.0	37.1
764.1379					926476	2.0	8.1	0.0	-5.0	0.0	-5.3	-8.0
766.1723					1954457	0.0	14.1	3.5	-11.8	-2.0	-14.5	-3.8
768.1655					1917468	0.0	18.6	0.0	-8.3	-1.8	-10.2	-15.2
770.1389					1218648	3.3	12.2	-1.2	-7.3	-6.5	-7.4	-7.4
771.1573					987677	4.8	6.7	-0.4	-6.2	-3.9	-6.1	-5.1
772.1819					827477	-0.5	3.8	1.7	-2.9	0.0	-4.6	0.0
773.4795					508239	0.0	1.7	0.0	-0.3	0.0	-1.8	-0.7
776.1540					740068	0.0	3.2	0.4	-2.9	-0.1	-3.7	-0.3
776.1540					537863	1.4	2.7	-0.1	-2.0	-0.4	-2.6	-2.1
779.1550					1230744	2.1	8.3	-0.4	-6.1	-1.3	-7.5	-4.1
782.1524					689782	0.0	3.6	0.3	-3.1	0.0	-3.2	-0.2
785.1686					2258765	-18.7	-19.0	-3.6	35.3	6.5	55.1	-8.1
786.1660					1110206	-11.2	-10.6	-2.6	23.8	2.6	31.9	-5.1
788.1739					5229874	8.2	23.9	1.3	-19.7	-5.5	-23.3	-15.8
789.1752					2589288	12.4	17.6	-3.0	-14.3	-5.8	-18.4	-15.5
790.1655					871352	1.2	4.6	0.0	-4.8	-0.9	-6.0	-0.1
791.1709					565710	0.0	3.0	0.0	-2.1	0.0	-3.0	0.0
792.4536	PS 34:3	C40H72NO10P	[M+Cl]-	-6.6	412872	0.0	1.3	-0.1	0.0	0.0	-0.5	-1.3
794.1727					1607274	0.1	12.4	0.4	-9.5	-2.8	-12.1	-2.3
795.1750					881931	1.5	7.8	-0.1	-4.1	-1.0	-6.0	-5.7
796.1693					563809	0.0	3.2	0.0	-2.4	-0.3	-3.3	-0.4
797.4366					589696	-7.7	-7.4	-0.8	12.6	28.2	0.5	-2.9
799.1638					1058759	0.6	4.7	2.0	-4.8	-1.3	-6.5	-0.8
802.1613					931368	4.4	6.1	-0.8	-4.9	-1.2	-6.1	-6.0
804.1438					1121667	3.5	8.5	-0.7	-6.2	-1.1	-7.9	-6.8
805.1476					604596	2.4	3.1	0.0	-2.6	-0.8	-3.5	-3.1
807.1528					559453	1.5	2.5	-0.3	-2.2	0.0	-2.8	-1.3
808.1601					432969	1.4	1.4	-0.1	-1.2	0.0	-1.9	-1.6
810.1541					4125477	0.0	19.2	6.2	-16.9	-3.5	-21.4	-3.8
811.1565					2138939	13.1	14.0	-3.7	-13.1	-5.3	-15.7	-13.7
813.1560					871385	0.1	6.2	0.0	-4.1	-0.8	-5.8	-1.2
816.1561					1816664	3.3	12.3	1.9	-11.3	-2.8	-12.9	-6.5
817.1576					879619	0.0	4.6	0.4	-4.2	0.0	-5.5	0.0

818.1521					812482	2.8	4.6	0.0	-3.9	-1.1	-4.5	-3.8
819.1642					565837	2.1	3.4	0.0	-2.7	-0.5	-3.0	-3.3
821.1456					780712	0.2	4.2	0.0	-3.8	-1.0	-4.5	0.0
821.6482	TG 47:3	C50H90O6	[M+Cl]-	6.2	1494564	0.8	9.4	3.2	-8.8	-4.0	-9.3	-2.5
826.1308					835762	2.2	5.3	0.0	-4.4	-2.7	-4.9	-2.8
830.1635					882232	5.2	4.5	-1.7	-4.4	-0.4	-5.5	-5.5
832.1380					3066321	11.7	20.0	-2.8	-17.1	-4.9	-21.0	-14.8
833.1445					1325124	3.8	9.7	-0.2	-8.0	-5.4	-9.8	-3.0
833.6660	TG 51:7	C54H90O6	[M-H]-	-0.6	438042	1.9	2.1	-0.3	-1.7	-0.7	-1.6	-2.3
838.1404					1131832	0.0	7.9	1.8	-6.7	-0.7	-7.7	-1.3
839.1419					633138	0.0	3.7	0.0	-2.5	0.0	-3.6	0.0
841.1537					866081	0.0	4.1	0.0	-3.4	0.0	-5.1	-0.3
844.1513					734868	2.6	4.1	-0.6	-2.7	-1.1	-4.2	-3.4
846.1311					611909	0.6	4.1	-0.2	-2.0	-0.8	-3.2	-1.9
848.1202					1043779	1.7	6.5	0.8	-6.0	-1.5	-7.5	-2.8
848.1202					652000	1.1	3.1	0.0	-3.0	-1.7	-2.9	-1.3
852.1487		C25H38N7O18P3S1	[M-H]-	5.4	805618	0.0	5.2	0.0	-4.2	-0.5	-5.2	0.0
854.1251					812677	0.0	3.9	1.0	-4.4	0.0	-4.6	0.5
855.1381					712570	2.5	4.0	-0.6	-3.2	-0.7	-4.3	-2.7
857.4536					378913	0.9	0.5	0.0	-0.5	0.0	-1.0	-1.3
860.1294					660288	2.5	4.3	0.0	-3.0	-0.5	-3.9	-4.4
861.1503					445691	0.8	0.7	0.0	-0.7	0.0	-1.9	0.0
863.1379					669403	0.0	4.7	0.0	-3.3	-0.6	-3.9	-0.4
866.1401					564796	0.0	1.6	0.3	-2.2	0.0	-2.4	0.0
868.1206					386192	0.0	0.2	0.0	-0.7	0.0	-1.0	0.0
872.1536	CoA 7:3	C28H42N7O17P3S	[M-H]-	4.4	2805861	3.2	15.0	4.1	-14.0	-3.6	-17.6	-5.9
873.1557					1536397	10.2	10.5	-2.3	-9.6	-3.3	-11.5	-12.0
874.1431					590893	0.0	2.7	0.0	-2.4	-0.1	-3.6	0.0
876.1148					711482	1.4	4.6	0.0	-3.6	-0.4	-3.9	-3.4
877.1216					494067	0.9	2.0	0.0	-1.3	-0.1	-1.9	-2.0
878.1457					468777	0.8	2.5	0.0	-1.7	-0.1	-1.8	-1.9
880.1436	CoA 5:1;O2	C26H42N7O19P3S	[M-H]-	4.5	405106	0.0	0.7	0.0	-0.8	0.0	-1.4	0.0
883.1425					679240	1.5	3.6	0.0	-3.6	-0.8	-4.5	-1.0
886.1444	CoA 5:0	C26H44N7O17P3S	[M+Cl]-	2.6	546568	1.3	2.2	-0.1	-2.0	-0.1	-2.9	-0.8
888.1278	CoA 4:0;O	C25H42N7O18P3S	[M+Cl]-	7.2	702071	2.0	4.4	0.0	-3.7	-0.8	-4.0	-3.4
889.1323					385014	0.0	0.7	0.0	-0.7	0.0	-0.9	-0.2
891.1331					437686	1.9	1.0	-0.1	-1.3	-0.2	-2.1	-1.2
891.4512					409584	0.7	0.0	0.0	-1.3	-0.2	-2.0	1.6
894.1376					2409784	12.8	15.0	-1.7	-14.2	-4.7	-16.6	-15.2
895.1398					1183219	6.0	7.3	-2.0	-7.0	-2.6	-8.6	-5.1
897.1375					461372	0.0	1.2	0.0	-0.6	-0.2	-2.0	0.0
900.1376					699284	0.3	4.0	0.0	-3.0	-0.5	-3.9	-0.6
901.1374					541993	2.6	2.3	-0.5	-1.8	-0.5	-2.9	-2.6
902.1318	CoA 5:0;O	C26H44N7O18P3S	[M+Cl]-	-5.9	495019	1.9	1.3	0.0	-1.7	0.0	-2.4	-1.2
903.1472	2-amino-5-oxocyclohex-1-enecarbonyl-CoA	C28H43N8O18P3S	[M-H]-	-9.3	485829	2.0	1.9	0.0	-1.8	0.0	-2.0	-2.7
905.1258					550061	2.0	2.1	-0.6	-2.3	-0.4	-2.9	-1.1
906.1382					436367	0.6	2.4	0.0	-1.3	0.0	-1.7	-1.8
908.1287	CoA 7:3	C28H42N7O17P3S	[M+Cl]-	2.4	528924	1.5	2.5	0.0	-2.0	0.0	-2.6	-2.5
910.1163					575822	2.0	2.8	0.0	-2.2	0.0	-2.9	-3.0
911.1269					365395	0.0	1.1	0.0	-0.6	0.0	-1.2	0.0
912.1340					336718	0.0	1.2	0.0	-0.5	0.0	-0.8	-0.9
914.1395	CoA 6:1;O	C27H44N7O18P3S	[M+Cl]-	2.6	700914	4.6	3.3	-0.8	-3.5	-0.5	-4.3	-4.9
916.1164	CoA 5:1;O2	C26H42N7O19P3S	[M+Cl]-	0.1	1069682	1.9	5.9	0.0	-5.8	-1.7	-7.3	-0.5
917.1282					632045	2.7	2.2	0.0	-2.9	-0.6	-3.0	-2.6
918.3570					496621	0.5	2.3	0.0	-1.1	0.0	-2.1	-1.9
920.1097					645002	0.0	2.5	0.2	-2.6	0.0	-3.3	0.0
923.1268					479723	1.4	2.4	0.0	-1.7	-0.2	-2.2	-1.9
925.1348					652046	2.9	2.5	0.0	-3.1	-0.5	-3.4	-3.0
929.3455					669076	0.3	3.9	0.0	-2.6	0.0	-3.8	-1.0
930.6872					629617	-0.3	4.0	0.0	-2.4	0.0	-2.8	0.0
932.1110	CoA 5:1;O3	C26H42N7O20P3S	[M+Cl]-	-0.2	402617	0.6	0.6	0.0	-1.0	0.0	-1.1	-0.5
934.1544	CoA 8:3;O3	C29H44N7O20P3S	[M-H]-	4.5	1059041	1.2	5.6	0.5	-5.7	-0.6	-7.0	-1.9
935.1518					647826	4.0	2.4	-0.8	-2.6	-0.9	-3.4	-3.6
936.1299					587967	0.2	2.7	0.0	-2.4	-0.1	-2.9	-1.0
938.1051					742967	3.0	4.8	-0.1	-4.1	-1.2	-4.3	-4.1
940.3361					667023	0.0	5.1	0.0	-2.8	-0.7	-3.9	-1.0
940.8377					388269	0.0	1.6	0.0	-1.1	0.0	-1.3	-1.3
944.1105	CoA 6:2;O3	C27H42N7O20P3S	[M+Cl]-	-0.7	531072	0.9	1.8	0.0	-1.9	0.0	-2.3	-1.4
945.1310					402322	0.8	1.2	0.0	-1.1	0.0	-1.5	-1.2
947.1200					396001	0.2	0.4	0.0	-1.1	0.0	-1.2	-0.1
948.6434					281077	0.0	0.0	0.0	0.0	0.0	-0.4	0.0
951.3263					509043	0.7	2.7	0.0	-1.5	0.0	-2.1	-2.4
954.1056					385307	1.5	1.0	0.0	-0.9	0.0	-1.0	-1.7
956.1365					1209102	2.0	7.4	0.0	-6.6	-2.0	-8.8	-2.3
957.1370					658972	0.8	1.8	0.2	-2.9	0.0	-3.6	0.0

959.1297					478016	2.6	0.3	-0.8	-0.8	-0.1	-0.9	-1.9
960.3464					469655	0.2	3.1	0.0	-1.2	0.0	-2.3	-1.6
961.1039					327668	0.0	0.8	0.0	-0.7	0.0	-0.8	0.0
962.1292					372844	0.0	0.8	0.0	-0.5	0.0	-0.8	0.0
964.1248	CoA 9:5;O2	C30H42N7O19P3S	[M+Cl]-	8.8	358002	0.0	0.7	0.0	-0.4	0.0	-1.2	0.0
967.6287	PI 44:5	C53H93O13P	[M-H]-	0.6	466486	1.3	1.8	-0.6	-1.3	-0.3	-1.9	-1.2
971.3368					527740	0.0	2.1	0.0	-1.5	0.0	-2.2	-0.3
972.1116					417578	0.2	0.5	0.0	-0.8	0.0	-1.2	-0.6
973.1142					331635	0.9	0.7	0.0	-0.8	0.0	-1.0	-0.7
975.1152					348560	0.0	0.7	0.0	-0.5	0.0	-0.9	0.0
976.1098					957460	3.2	7.2	-0.6	-5.8	-2.3	-7.6	-3.3
976.1098	CoA 6:2;O5	C27H42N7O22P3S	[M+Cl]-	8.9	308244	0.0	0.5	0.0	-0.2	0.0	-0.6	0.0
981.8262					479153	0.6	2.2	0.0	-0.3	0.0	-1.5	-2.4
982.6222					333791	0.3	0.7	0.0	-0.4	0.0	-0.6	-0.9
984.1182					427924	0.3	1.0	0.0	-1.6	0.0	-1.6	-0.3
987.1273					384223	0.0	1.5	0.0	-0.6	-0.1	-1.5	-0.2
989.1090					386764	1.4	0.8	0.0	-1.1	-0.1	-1.2	-1.3
990.1233					373695	0.4	1.4	0.0	-0.8	0.0	-1.3	-0.8
992.8167					404630	0.0	1.9	0.0	-0.6	-0.3	-1.5	-0.5
993.3183					412272	0.7	1.5	0.0	-1.4	0.0	-1.4	-1.4
994.0989					373008	0.5	1.0	0.0	-0.7	-0.1	-1.2	-0.4
995.1097					291955	0.0	0.5	0.0	0.0	0.0	-0.5	-0.1
996.1148					290636	0.1	0.2	0.0	-0.3	0.0	-0.4	-0.3
998.1174					432904	0.0	1.3	0.0	-1.2	0.0	-1.6	0.0
1000.1023					588526	1.2	2.5	0.0	-2.0	0.0	-2.6	-2.2
1001.1148					420614	0.0	1.6	0.0	-1.2	0.0	-1.4	0.0
1002.3375					389225	0.1	1.7	0.0	-0.4	0.0	-1.7	-1.0
1004.0933					266900	0.0	0.0	0.0	-0.2	0.0	-0.4	0.0
1006.1070					412592	0.5	1.5	0.0	-1.1	0.0	-1.3	-1.3
1007.1111					314128	0.0	0.1	0.0	-0.4	0.0	-0.8	0.0
1009.1143					463293	1.8	1.8	0.0	-1.7	-0.4	-1.9	-2.3
1013.3272					441194	0.0	1.1	0.0	-1.3	0.0	-1.4	-0.4
1014.0961					340030	0.0	0.8	0.0	0.0	0.0	-1.0	0.0
1015.1008					277739	0.4	0.1	0.0	-0.5	0.0	-0.3	-0.1
1018.1299					497240	0.8	1.1	0.0	-1.5	0.0	-2.0	-0.9
1020.1092					497240	0.2	1.2	0.0	-1.2	0.0	-1.4	-0.1
1024.3182					516805	0.3	2.1	0.0	-1.3	0.0	-2.2	-0.8
1024.8197					315409	0.0	0.9	0.0	-0.3	0.0	-1.0	0.0
1028.0957					403194	0.0	1.5	0.0	-0.6	-0.1	-1.5	0.0
1029.1124					342735	0.4	1.0	0.0	-1.0	0.0	-0.9	-0.9
1030.1006					346144	0.7	0.4	0.0	-0.6	0.0	-0.9	-0.7
1031.1006					312220	0.0	0.3	0.0	-0.4	0.0	-0.5	-0.3
1035.3086					395613	0.7	1.7	0.0	-0.7	-0.1	-1.6	-0.9
1035.8106	TG 63:8	C66H112O6	[M+Cl]-	-4.5	271020	0.0	0.4	0.0	-0.1	0.0	-0.5	0.0
1040.1162					762821	1.8	3.3	0.0	-3.4	-1.6	-4.8	-0.7
1041.1153					457100	0.5	0.3	0.0	-1.1	0.0	-1.5	0.0
1044.0812					394232	0.1	0.9	0.0	-0.9	0.0	-1.3	0.0
1045.0854					341218	0.5	1.0	0.0	-0.6	0.0	-1.0	-0.8
1046.1068					330366	0.6	0.0	0.0	-0.6	0.0	-0.3	-0.4
1047.1069					321960	1.4	0.0	0.0	-0.3	0.0	-0.7	-0.5
1048.1048					301118	0.0	0.9	0.0	-0.2	0.0	-0.7	0.0
1051.0997					387810	0.9	0.6	0.0	-0.8	0.0	-1.1	-1.1
1054.1024					364594	1.0	0.6	0.0	-0.7	0.0	-0.8	-1.1
1055.3177					391761	0.3	1.4	0.0	-0.7	-0.1	-1.7	-0.8
1056.0916					311746	0.6	0.0	0.0	-0.6	0.0	-0.5	0.0
1057.0949					304678	0.5	0.0	0.0	0.0	0.0	-0.6	-0.9
1059.0960					319098	0.3	0.7	0.0	-0.5	0.0	-0.9	-0.1
1060.0913					296223	0.0	1.2	0.0	-0.2	0.0	-0.7	0.0
1062.0984					662643	3.2	3.3	-0.6	-3.3	-1.2	-4.2	-2.3
1063.1027					378793	0.2	0.5	0.0	-0.8	0.0	-1.0	0.0
1064.0935					288809	0.2	0.4	0.0	-0.1	0.0	-0.4	-0.4
1065.8043					393853	0.7	1.1	0.0	-0.9	0.0	-1.2	-1.4
1066.8072					281684	0.0	0.6	0.0	-0.2	0.0	-0.4	0.0
1068.0980					364475	0.9	0.4	0.0	-0.6	-0.1	-1.1	0.0
1069.0962					304686	0.0	0.4	0.0	0.0	0.0	-0.7	0.0
1071.6122					303116	0.0	1.2	0.0	-0.2	0.0	-0.7	-0.1
1073.0883					340053	0.4	1.0	0.0	-0.8	0.0	-0.8	-1.1
1074.1007					316071	0.0	0.8	0.0	0.0	0.0	-1.0	0.0
1074.6099					261673	0.0	0.1	0.0	0.0	0.0	-0.1	-0.2
1077.2984					261673	1.3	0.4	0.0	-0.8	0.0	-1.1	-1.1
1078.0829					314532	0.0	0.1	0.0	0.0	0.0	-0.6	0.0
1079.0923					304973	0.4	0.2	0.0	-0.4	0.0	-0.7	-0.4
1080.1005					252017	0.0	0.0	0.0	0.0	0.0	-0.1	-0.4
1082.0974					360538	0.0	1.1	0.0	-0.7	0.0	-0.8	-0.6
1084.0790					380290	0.4	0.9	0.0	-0.9	0.0	-1.0	-1.0
1085.0924					353169	0.1	0.7	0.0	-0.6	-0.3	-1.4	0.0

1085.6002					353169	0.5	0.4	0.0	-0.2	0.0	-0.6	-0.3
1088.2904					287063	0.0	0.8	0.0	0.0	0.0	-0.7	0.0
1090.0987					354717	0.9	0.0	0.0	-0.2	0.0	-0.5	0.0
1093.0928					371784	0.0	0.0	0.0	-0.5	0.0	-0.9	0.0
1094.0884					266641	0.0	0.0	0.0	-0.1	0.0	-0.6	0.0
1095.0807					229909	0.0	0.0	0.0	-0.1	0.0	0.0	0.0
1097.3074					390843	0.5	1.3	0.0	-1.1	0.0	-1.3	-0.9
1099.0934					256680	0.0	0.0	0.0	0.0	0.0	-0.3	0.0
1102.1087					461792	1.1	1.2	0.0	-1.1	-0.2	-2.2	-0.1
1104.0914					346720	0.5	0.0	0.0	-0.7	0.0	-0.8	0.0
1108.2974					398670	0.8	0.9	0.0	-0.6	0.0	-1.5	-0.5
1109.3001					2184581	0.0	11.1	0.0	-4.8	-3.2	-10.3	-1.3
1110.5338					1344466	-1.7	10.3	0.0	-3.2	-2.3	-8.6	-0.5
1111.5487					624212	-0.1	2.1	0.0	0.0	-0.6	-2.2	0.0
1113.0943					304156	0.0	0.2	0.0	-0.1	0.0	-0.4	0.0
1114.0853					333508	0.1	0.4	0.0	-0.1	0.0	-0.7	0.0
1116.0905					335020	0.3	0.3	0.0	0.0	0.0	-0.4	-0.2
1118.0849					302227	0.0	0.0	0.0	-0.1	0.0	-0.3	0.0
1119.2880					353711	0.3	0.9	0.0	-0.5	0.0	-0.9	-0.4
1120.0789					292584	0.9	0.0	0.0	-0.2	0.0	-0.3	-0.2
1121.0829					269542	0.0	0.0	0.0	0.0	0.0	-0.4	0.0
1121.0829					253736	0.0	0.3	0.0	0.0	0.0	-0.5	0.0
1124.0941					489845	1.1	1.5	0.0	-1.7	-0.1	-2.0	-1.0
1125.0939					370276	0.8	0.0	0.0	-0.2	-0.3	-1.0	0.0
1127.0881					319411	0.0	0.0	0.0	0.0	0.0	-0.6	0.0
1130.0873					302436	0.4	0.4	0.0	-0.2	0.0	-0.5	-0.7
1131.5117					3075924	0.6	25.6	-2.0	-13.2	-5.3	-17.1	-13.0
1132.5142					1944331	0.2	14.5	2.7	-9.9	-0.9	-11.6	-9.1
1133.5186					817387	-1.6	7.1	0.0	-2.8	-1.3	-5.0	0.0
1135.0816					360808	0.3	1.4	-0.2	-0.7	-0.2	-1.3	-0.2
1136.0835					294543	1.0	0.0	0.0	-0.2	0.0	-0.6	-0.1
1139.2955					341555	0.4	0.6	0.0	-0.5	0.0	-0.8	-0.7
1140.0735					287799	0.0	0.2	0.0	0.0	0.0	-0.6	0.0
1141.0786					270126	0.4	0.0	0.0	0.0	0.0	-0.4	0.0
1141.0786					227985	0.0	0.2	0.0	0.0	0.0	-0.2	0.0
1143.0755					294806	0.1	0.0	0.0	0.0	0.0	-0.3	0.0
1146.0790					408364	0.7	0.3	0.0	-1.0	0.0	-1.1	-0.2
1147.0837					259969	0.2	0.0	0.0	-0.1	0.0	-0.4	0.0
1150.2875					350941	0.2	0.3	0.0	-0.4	0.0	-1.1	0.0
1153.4923					2584207	5.1	17.9	-2.3	-12.0	-3.1	-15.0	-11.3
1154.4952					259969	-1.1	14.7	0.5	-9.0	-2.5	-11.8	-1.9
1155.4987					668074	0.0	5.7	0.0	-2.9	-0.4	-3.7	-2.9
1158.0790					285534	0.2	0.4	0.0	-0.3	0.0	-0.8	0.0
1160.2803					322703	0.0	0.3	0.0	0.0	0.0	-0.3	-0.5
1162.0660					284950	0.5	0.3	0.0	-0.3	0.0	-0.7	0.0
1163.0710					244350	0.0	0.2	0.0	-0.1	0.0	0.0	0.0
1166.0762					329895	1.2	0.0	0.0	-0.5	0.0	-0.8	-0.4
1167.0744					282816	0.0	0.0	0.0	0.0	0.0	-0.4	0.0
1169.0734					298189	0.4	0.4	0.0	-0.6	0.0	-0.8	0.0
1170.0751					240946	0.1	0.0	0.0	-0.1	0.0	-0.4	0.0
1173.2834					274969	0.0	0.0	0.0	0.0	0.0	-0.2	0.0
1174.0679					308595	0.3	0.1	0.0	-0.2	0.0	-0.4	0.0
1175.4726					1648547	1.2	11.6	0.2	-7.1	-2.5	-11.5	-5.1
1176.4760					1111825	0.0	10.6	0.0	-5.7	-2.5	-8.9	-2.1
1181.2850					281756	0.0	0.0	0.0	0.0	0.0	-0.1	0.0
1183.7728					236267	0.0	0.0	0.0	0.0	0.0	-0.1	0.0
1186.0860					417377	1.5	0.6	-0.5	-1.0	0.0	-1.6	-0.1
1188.0710					296550	0.1	0.5	0.0	-0.4	0.0	-1.0	0.0
1190.0633					246102	0.0	0.0	0.0	0.0	0.0	0.0	0.0
1192.2760					342768	0.0	1.2	0.0	-0.6	0.0	-0.9	0.0
1192.7714					273649	0.0	0.2	0.0	0.0	0.0	-0.4	0.0
1194.7653					232821	0.0	0.4	0.0	0.0	0.0	-0.1	0.0
1197.4546					781498	0.0	8.4	0.0	-4.0	-1.3	-5.7	-2.6
1198.2646					509507	0.0	3.4	0.0	-1.9	0.0	-2.5	-1.6
1198.7551					258808	0.0	0.2	0.0	-0.3	0.0	-0.3	0.0
